# The Forest After Tomorrow: Projecting the Impact of a Collapsing Atlantic Meridional Overturning Circulation on European Tree‐Species Distributions

**DOI:** 10.1111/gcb.70185

**Published:** 2025-04-24

**Authors:** Sina Heubel, Anja Rammig, Allan Buras

**Affiliations:** ^1^ Land Surface‐Atmosphere Interactions TU Munich Freising Germany; ^2^ Department of Environmental Systems Science Institute of Terrestrial Ecosystems, ETH Zurich Zurich Switzerland

**Keywords:** climate envelope models, CMIP6, quantile mapping, thermohaline circulation, tipping point, tree‐species richness

## Abstract

Forest tree species are expected to experience a substantial redistribution due to climate change. While previous work has emphasized the effects of a warmer and drier climate on European tree‐species distributions, to date no study has investigated the potential impact of a collapse of the Atlantic Meridional Overturning Circulation (AMOC). Here, we deploy climate‐envelope models to quantile mapped, high‐resolution (1km^2^) CMIP6 climate projections and compare tree‐species distributions under an active AMOC vs. an inactive AMOC scenario. Across Europe, our tree‐species projections indicate contrasting impacts of the two scenarios. In Scandinavia, many of the currently abundant tree species were projected a dramatic decline and partial disappearance due to the strong cooling under an inactive AMOC. In Central and Southern Europe, however, some of the currently abundant species suffered less under an inactive AMOC compared to an active AMOC scenario while others—such as the economically important species of Norway spruce—almost went extinct. As opposed to the classic climate‐change scenario supporting Mediterranean species in Central Europe, projected European tree‐species portfolios consisted of a higher share of boreal, cold‐tolerant species in the inactive AMOC scenario. Finally, tree‐species diversity was projected to decline even stronger under an inactive vs. an active AMOC scenario. Altogether, while an AMOC collapse may locally result in more favorable conditions for specific species in comparison to a classic climate‐change scenario, the dramatic economic and ecological consequences suggested by our projections indicate the urgent need for climate‐change mitigation to lower the likelihood of an AMOC collapse.

## Graphical abstract

This study examines the effects of a collapsing Atlantic Meridional Overturning Circulation (AMOC) in comparison to a common climate‐change scenario. It shows that an AMOC collapse would result in contrasting tree‐species portfolios across Europe in comparison to an active AMOC scenario and suggests catastrophic effects on forest ecosystems in Scandinavia.

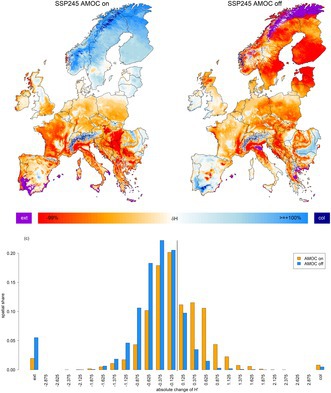

## 1 Introduction

The climate‐change induced increasing frequency and magnitude of so‐called hotter droughts has resulted in widespread forest decline and tree dieback (Allen et al. 2015; Anderegg et al. 2015; Buras et al. 2020, 2018; Cailleret et al. 2017; Orth et al. 2016; Schuldt et al. 2020; Senf et al. 2020). As a consequence, tree‐species distributions will undergo vast changes in the 21st century, depending on how the climatological boundaries under which they survive, sometimes termed their climate envelope, are realized (Buras and Menzel 2019; Chakraborty et al. 2021; Dyderski et al. 2018; Koch et al. 2022; Martes et al. 2024; Walentowski et al. 2017). Therefore, local tree‐species portfolios are likely to change, posing a general threat to forest integrity and a huge challenge to forestry stakeholders, who urgently seek to identify the most robust tree species for the ongoing forest conversion aiming at climate‐change resilient forests (Buras and Menzel 2019; Martes et al. 2024; Walentowski et al. 2017; Wessely et al. 2024).

For the European continent, previous studies have mapped the potential shift of tree‐species distributions under various climate‐change scenarios based on CMIP5 multi‐model climate projections (Buras and Menzel 2019; Chakraborty et al. 2021; Dyderski et al. 2018; Koch et al. 2022; Mauri et al. 2022). In general, all of these studies projected several species to shift their distributional center northward, resulting in the decline of the widely abundant and economically important tree species of Norway spruce ( *Picea abies* L.) and Scots pine ( *Pinus sylvestris* ) in Central Europe, which were partly projected to be replaced by Mediterranean tree species. Moreover, a local study focused on the climate‐envelope exceedance frequency of specific Central European tree species under various CMIP6 projections, indicating similar detrimental effects of climate change on those two species (Martes et al. 2024).

While these studies provided valuable information for forestry stakeholders, they did not consider the possibility of a collapse of the Atlantic Meridional Overturning Circulation (AMOC). Already nowadays, AMOC features its weakest state over the last millennium (Caesar et al. 2021) likely induced by an enhanced freshwater inflow from the Greenland ice‐sheet (Rahmstorf 2006; Rahmstorf et al. 2015; Rahmstorf and Ganopolski 1999). While some studies reported an AMOC collapse within the 21st century to be unlikely (Baker et al. 2025; Masson‐Delmotte et al. 2021), several studies independently identified early‐warning indicators in the North Atlantic suggesting an increasing risk of an AMOC collapse in the second half of the 21st century (Boers 2021; Ditlevsen and Ditlevsen 2023; Michel et al. 2022; Rahmstorf 2024; van Westen et al. 2024). As a consequence of an AMOC collapse, the climate across the European continent would become significantly cooler and drier (Jackson et al. 2015; Rahmstorf 2006; Rahmstorf and Ganopolski 1999), thus partially counteracting the anticipated climate‐change induced warming albeit under drier conditions. Since this would directly affect the realization of climate envelopes, tree‐species distributions and the resulting portfolios are likely to differ from the tree‐species projections provided thus far (e.g., Buras and Menzel 2019; Chakraborty et al. 2021; Dyderski et al. 2018; Martes et al. 2024; Mauri et al. 2022). Yet, no study has provided insights into the potential shifts in tree‐species distributions under an AMOC collapse, and it remains an open question whether an AMOC collapse may have more positive or negative effects on Europe's forests given a partial mitigation of warming vs. a reduction of precipitation.

To overcome this research gap, we here present projections of tree‐species distributions under three different CMIP6 climate‐change scenarios (SSP1‐2.6, SSP2‐4.5, SSP5‐8.5) at high spatial resolution (1 km^2^). For all selected scenarios, we moreover incorporated an AMOC‐collapse scenario by superimposing the effect of an AMOC collapse on temperature and precipitation onto the CMIP6 climate projections. In our evaluation, we focus on the differences between two AMOC modes, active vs. inactive, emphasizing the following questions:

- Q1: How are the simulated occurrence probability as well as spatial distribution of Europe's currently most abundant forest tree‐species affected?
- Q2: How is the current appearance of Europe's forests—represented by locally dominant species—projected to change?
- Q3: Which species are likely to substitute locally extinct tree species?
- Q4: How is the tree‐species diversity of Europe's forests affected?

By addressing these questions, we seek to identify vulnerable species as well as potential hot‐spots of forest decline under all scenarios and to elucidate the potential impacts of an AMOC collapse.

## 2 Material and Methods

### 2.1 Input Data

#### 2.1.1 Statistical Downscaling of Historical Observations

In terms of model calibration (section 2.2) and projecting the historical reference for tree‐species distributions, we used the gridded temperature and precipitation data from the climate research unit (CRU TS version 4.07) at a spatial resolution of 0.5° and a monthly temporal resolution (Harris et al. 2020). These data were cropped and masked to the extent of continental Europe, including Great Britain and Ireland, and truncated to the period 1951–2020. Since the relatively coarse spatial resolution of 0.5° does not allow for resolving elevational effects on tree‐species distributions reliably (Buras and Menzel 2019), we applied a statistical change‐factor downscaling using high‐resolution temperature and precipitation monthly climatology (CHELSA version 2.1, Karger et al. 2023). Details of this downscaling are provided in Appendix A.1. An example of the gain in precision by improving the spatial resolution is provided in Figure S1.

#### 2.1.2 Quantile Mapping of CMIP6 Projections

To allow for projections of potential tree‐species distributions under historic and future climate conditions, we retrieved monthly temperature means and precipitation sums from ten models in the CMIP6 ensemble (see Table S1) for the historic period of 1951–2014 as well as the period 2015–2100 considering the emission scenarios SSP1‐2.6, SSP2‐4.5, and SSP5‐8.5. The model selection was based on the availability of temperature and precipitation data for all scenarios by the time the data were retrieved (August 2022). In order to spatially downscale CMIP6 projections, we applied a quantile mapping approach (Ekström et al. 2015). Details of the quantile mapping are provided in Appendix A.2. A visual impression of the quantile‐mapping success is presented in Figure S2. Due to the large computational demand of the quantile mapping, it was implemented in the ‘Rcpp’‐package, which provides an efficient ‘R’‐interface to C++ (Eddelbuettel and François 2011).

#### 2.1.3 AMOC Collapse Scenarios

To obtain projections of temperature and precipitation which resemble an AMOC collapse, we extracted information from figures 4 and 7 presented in Jackson et al. (2015). These figures represent surface air temperature anomalies (SAT) and relative precipitation anomalies (RPA) of a simulation using a high‐resolution global circulation model (HadGEM3) that was perturbed with a large freshwater forcing (100 Sverdrup evenly distributed over 10 years) in the North Atlantic, which resulted in an AMOC collapse (for details, see Jackson et al. 2015). We chose this study for data extraction, since it provides detailed maps of temperature and precipitation changes across most of our study region in Europe. Details on the extraction of the corresponding information are provided in Appendix A.3. To quantify the representativity of the derived maps regarding the original data, we provide a visual comparison which also depicts the overall high correlations (0.87 ≤ *r* ≤ 0.93) between original and derived data (Figure S3).

The obtained monthly SAT and RPA maps (Figure S4) were superimposed on the quantile mapped temperature and precipitation projections for all three considered scenarios to represent the full spectrum of potential impacts (section 2.1.4). Considering the period of superimposition, we selected the very last climate normal period in the 21st century, that is, 2071–2100 (see section 2.1.4). The selection of this period is in line with Ditlevsen and Ditlevsen (2023), who, based on an extrapolation of observations and by means of early‐warning signals, estimated an AMOC collapse to occur around the year 2060. Taken together, we eventually obtained three AMOC‐collapse scenarios representative of the SSP1‐2.6, SSP2‐4.5, and SSP5‐8.5 projections in the period 2071–2100 by superimposing anomalies of surface air temperature and precipitation representative of an AMOC collapse.

#### 2.1.4 Bioclimatic Parameters

From the historic and future climate projections of temperature and precipitation, we derived 17 bioclimatic variables. Since tree physiology is strongly determined by ambient temperature and plant water availability, these 17 variables were related to seasonal integrations of temperature and climatic water balance (CWB), that is, the difference between precipitation and potential evapotranspiration (Thornthwaite 1948; Zang et al. 2020). Selection of sub‐annual periods partly accounts for the effects described in Martes et al. (2024) who claimed annual climatology to mask the impacts of extremes on tree‐species distributions. In addition, we for both temperature and CWB provided two parameters resembling continentality which we either defined as the standard deviation or the range of monthly values over the annual cycle. All derived parameters were averaged for each grid cell over two historic climate normal periods (1951–1980 in the following ‘early historic period’, 1991–2020 in the following ‘late historic period’) and three future periods (2021–2050, 2046–2075, 2071–2100) for the 3 CMIP6 scenarios and for the period 2071–2100 for the 3 AMOC‐collapse scenarios, resulting in altogether 14 projection periods (2 historic, 9 standard CMIP6, 3 AMOC‐collapse scenarios). An overview on the bioclimatic parameters is presented in Table S2.

#### 2.1.5 Tree‐Species Observations

For the calibration of the climate envelopes needed for projecting tree‐species distributions, we used a compiled European national forest inventory dataset (NFI) provided in Mauri et al. (2022). This dataset holds 589,937 occurrences of altogether 69 tree species distributed across most of Europe. Since the reliability of derived climate envelopes largely depends on the sample size of the underlying data, we selected those from the 69 tree species which provided at least 5900 records (i.e., 1% share of all data), ending up with 26 tree species that in total made up 87.8% of the whole dataset, i.e., 517,924 observations.

When inspecting these data prior to the model development, we realized that some species occurrences did not mirror all actual occurrences. This was particularly the case for Norway spruce (*Pices abies* L.) in Central Europe, which—due to its economic importance—frequently has been planted outside its natural range. As a consequence, species projections based on these data underrepresented the actual occurrence of Norway spruce in Central Europe and therefore provided less representative results. To overcome this, we included an additional source of information, namely the species occurrence data as provided by the German national forest inventory (Bundeswaldinventur, BWI) which is freely available at a spatial precision of 1 km^2^ (https://bwi.info/Download/de/BWI‐Basisdaten/). From this dataset, we added occurrence records for the three most abundant tree species in Germany, that is, Norway spruce, Scots pine ( *Pinus sylvestris* L.), and European beech ( *Fagus sylvatica* L.). While the addition of occurrence data for European beech likely did not alter the eventual climate envelope for this species due to large congruency between NFI and BWI, the addition of data for Scots pine and particularly Norway spruce resulted in a stronger representation of lowland occurrences, thus more robustly representing the actual current distribution of these widely abundant species (Figure S5). To avoid pseudo‐replicates introduced by duplicates, we only selected unique coordinate pairs. Adding the BWI data to the truncated NFI data resulted in a total of 544,910 observations used for the development of climate envelopes. An overview on the 26 selected tree species and their relative share of observations is provided in (Figure S6). For model validation (see section 2.2.), we moreover retrieved chorological maps for these 26 tree species (Caudullo et al. 2017). However, since no chorological map was available for *Robinia pseudoacacia* we excluded this species from our analyses.

### 2.2 Climate‐Envelope Models

To project tree‐species distribution based on the bioclimatic parameters, we defined climate envelopes for each species, based on conflated probability‐density functions of climate parameters. In brief, the approach identifies 2–3 best‐fit bioclimatic parameters for each species that were selected based on verification skills leaning on Sensitivity, Specificity, and True Skill Statistics (TSS) (Allouche et al. 2006; Yang et al. 2022). Species‐specific probability density functions of these parameters were conflated using Fisher's combined probability test (Fisher 1970). Thereby, we generated climate‐envelope models for altogether 24 different species (one species failed the validation) with good (*n* = 19) or excellent predictive performance (*n* = 5). Finally, we applied the climate‐envelope models to project the future occurrence probability (*p*) of these 24 tree species under the 14 different scenarios (see section 2.1.4) for each of the ten CMIP6 models (section 2.1.2). Eventually, the scenario‐specific projections of occurrence probability *p* were averaged over all models to represent an ensemble mean. Note that since these projected occurrence probabilities *p* rely on climate envelopes, they can also be considered a measure of climatic habitat suitability. A detailed description of this approach and corresponding validation skills is provided in Appendix A.4 and Table S3, respectively. Moreover, Table S4 provides a report on model details in accordance with a recently proposed standard protocol for reporting ODMAP (Overview, Data, Model, Assessment, and Prediction) of species distribution models (Zurell et al. 2020).

Initially we also implemented the reproducibility scripts provided in Mauri et al. (2022), which allow for computing an ensemble of species‐distribution models with the BIOMOD2 package (Thuiller et al. 2009). Moreover, we applied the concept of climate analogues as utilized in Buras and Menzel (2019). However, both methods provided partly unrealistic (SDM ensemble) or patchy (analogues) and therefore suboptimal projections. In particular, the SDM ensemble approach resulted in future projections of several species outside their historic climate envelope. That is, some species were projected future occurrences at e.g., higher mean annual temperatures. A prominent example of this mismatch of historic and projected climate envelope is shown for *Picea abies* (Figure S7), while other species were affected, too. Because of this inconsistency, we eventually opted for using climate envelopes since they achieved good to excellent verification statistics, by definition remain consistent over time (see also Figure S7), and therefore more realistically reflect the observed decline of several species in Central Europe such as Norway spruce or Scots pine under current and future conditions.

### 2.3 Statistical Evaluation

Given the multitude of selected species (24) and scenarios (14) which result in a total of 336 ensemble projections, our statistical evaluation emphasized the four most abundant tree species—Scots pine ( *Pinus sylvestris* L.), Norway spruce ( *Picea abies* L.), European beech ( *Fagus sylvatica* L.), and Common oak ( *Quercus robur* L.)—as well as the climate scenario with the currently highest likelihood of realization (SSP2‐4.5 with AMOC on and off, respectively) at the end of the 21st century (2071–2100). Projections for all other species and scenarios are provided in an online repository (see data availability statement) and can moreover be visualized and downloaded in an interactive shiny‐app (http://app.forestmonitoringhub.eu).

In terms of evaluation, we firstly calculated the changes of *p* between the early historic period and the future projections, thereby addressing research question Q1. In particular, we computed the relative change in *p* between any of the future and historic periods.

(1) $$\mathrm{\delta p}=\left(\frac{p_{\text{future}}}{p_{\text{historic}}}-1\right)∙100\%$$

To specifically emphasize grid cells where a given tree species was projected to (1) become extinct or (2) expand into a new habitat, values of *δp* equaling −100% (extinction) or infinite (expansion) were highlighted with specific colors in the corresponding maps. Projections for the early and late historic period as well as their *δp* are shown in Figure S8 to visualize that the tree‐species projections for the historic period match actual distributions well and to exemplify the computation of *δp*.

To reflect changes in the actual distribution of a species (Q1), we computed a weighted geometric mean across coordinates representative of a species' distribution center under the various scenarios. That is, the longitude and latitude coordinates of each grid cell were considered for the geometric mean computation and weighed according to the actual occurrence probability. Due to the weighting, grid‐cells featuring an occurrence probability of zero were not considered, while those with an occurrence probability of 1 received the strongest weight. These distribution centers were mapped for each species and scenario to visualize the spatial change of its distribution center as an indicator of climate‐change induced relocation of the climate envelope. The size of the symbols used to indicate the distributional centers was scaled according to the scenario‐ and period‐specific spatial extent of a given species. Distribution centers and spatial extents were compared for the four focal species among all 14 scenarios.

Moreover, as an additional representation of species distribution changes, we computed the mean area‐weighted occurrence probability for each period and scenario over all scenario‐specific grid cells in which the species was projected to occur. These mean occurrence probabilities were then visualized as time‐series to reflect change trajectories under the different scenarios. In combination, the weighted mean coordinates and spatial extent together with the change trajectories of mean occurrence probabilities allow for visualizing the general performance of any of the focal species under the considered scenarios as well as the comparison among them (Q1).

In a next step, we computed *δp* (equation 1) for the species which was projected to have the locally highest occurrence probability under the early historic period (now considering all 24 species) mirroring the degree to which a local forest may change under climate change (Q2). If the species with the historically highest *p* were to feature a strong decline under climate change, this would likely indicate a remarkable change in the species composition of the affected forests. As with the species specific *δp* maps, we highlighted grid cells in which the historically dominant species went extinct. In a similar manner, we computed *δp* for the species that was projected to have the locally highest occurrence probability under any of the future scenarios. This was done to indicate whether the species being projected to have the locally highest future occurrence actually suffered (decline in *p*) or benefitted (increase) from climate change (Q2). Again, regions which were newly colonized by a species (i.e., only occurring in future scenarios and not occurring under historic conditions) were highlighted, as were regions in which none of the 24 considered species was projected to occur.

Next, we generated alluvial plots for the four most dominant species to identify the species that were projected to replace the currently dominant species in case they locally went extinct (Q3). Since for each species a multitude of potential replacement species were identified, we again emphasized the four focal species pine, spruce, beech, and oak in our visualization as well as the most frequently observed replacement species with more than 20,000 replacements.

Finally, to represent the degree of change with respect to tree‐species diversity (Q4), we computed Shannon's biodiversity index H' for each grid‐cell (Shannon 1948):

(2) $$H^{\prime }=-\sum_{1}^{k}\left(\pi_{i}∙\log\left(\pi_{i}\right)\right)$$

where *k* refers to the number of species with *p* > 0 within a grid cell for a given scenario‐specific projection, *i* refers to the species, and π refers to the relative share of the *i*‐th occurrence probability *p* from all summed occurrence probabilities:

(3) $$\pi_{i}=\frac{p_{i}}{\sum_{i}^{k}p_{i}}$$

Here, a decrease in H' would indicate a projected decreasing tree‐species diversity within a given grid‐cell and thus, that the projected climate envelope provides a suitable habitat for fewer species. In the evaluation we therefore emphasized *δH'* which denotes the absolute change of *H′* between the early historic period and a given future scenario (Q4). To emphasize on grid cells where (1) forests disappear or (2) expand into a new habitat, values of *δH'* equaling zero (disappearance) or infinite (expansion) were highlighted in the corresponding maps.

In order to reflect the uncertainty of projections we conducted two additional analyses. In the first analysis, for each pixel, species, and scenario‐period combination we computed the percentual standard deviation of projected *p* across all ten models (j) in relation to the pixel‐specific mean *p* of these models:

(4) $$S_{p}=\frac{\sqrt{\frac{\sum_{j}^{n}(p_{j}-{\bar{p)}}^{2}}{n-1}}}{\bar{p}}∙100\%,$$

with

(5) $$\bar{p}=\frac{\sum_{j=1}^{n}p_{j}}{n},$$

and j = 1, …, 10 (models).

Thereby, we achieved species‐specific estimates on the uncertainty of projections for each scenario‐period combination. To map these uncertainties, we visualized mean and maximum percentual standard deviation over all 24 species for the focal scenario‐period combination of SSP2‐4.5 in the period 2071–2100 (Figure S9).

Secondly, to partition uncertainty into contributions from models vs. scenarios, we pursued the approach presented in Diniz‐Filho et al. (2009). That is, for each pixel we computed a two‐way ANOVA with model and scenario as factorial explanatory variables and derived the corresponding proportional sum of squares in relation to the total sum of squares (including residuals). Sum of squares related to model, scenario, and residual error were visualized in maps as well as in a boxplot for direct comparison (Figure S10). All analyses were conducted using ‘R’ (version 4.3.1) extended with the packages ‘terra’ (Hijmans et al. 2022) and ‘Rcpp’ (Eddelbuettel and François 2011). All files supporting the model development and application are available at an open‐data repository (see data availability statement).

## 3 Results

### 3.1 Projected Changes in Europe's Most Abundant Tree Species (Q1)

**FIGURE 1:**
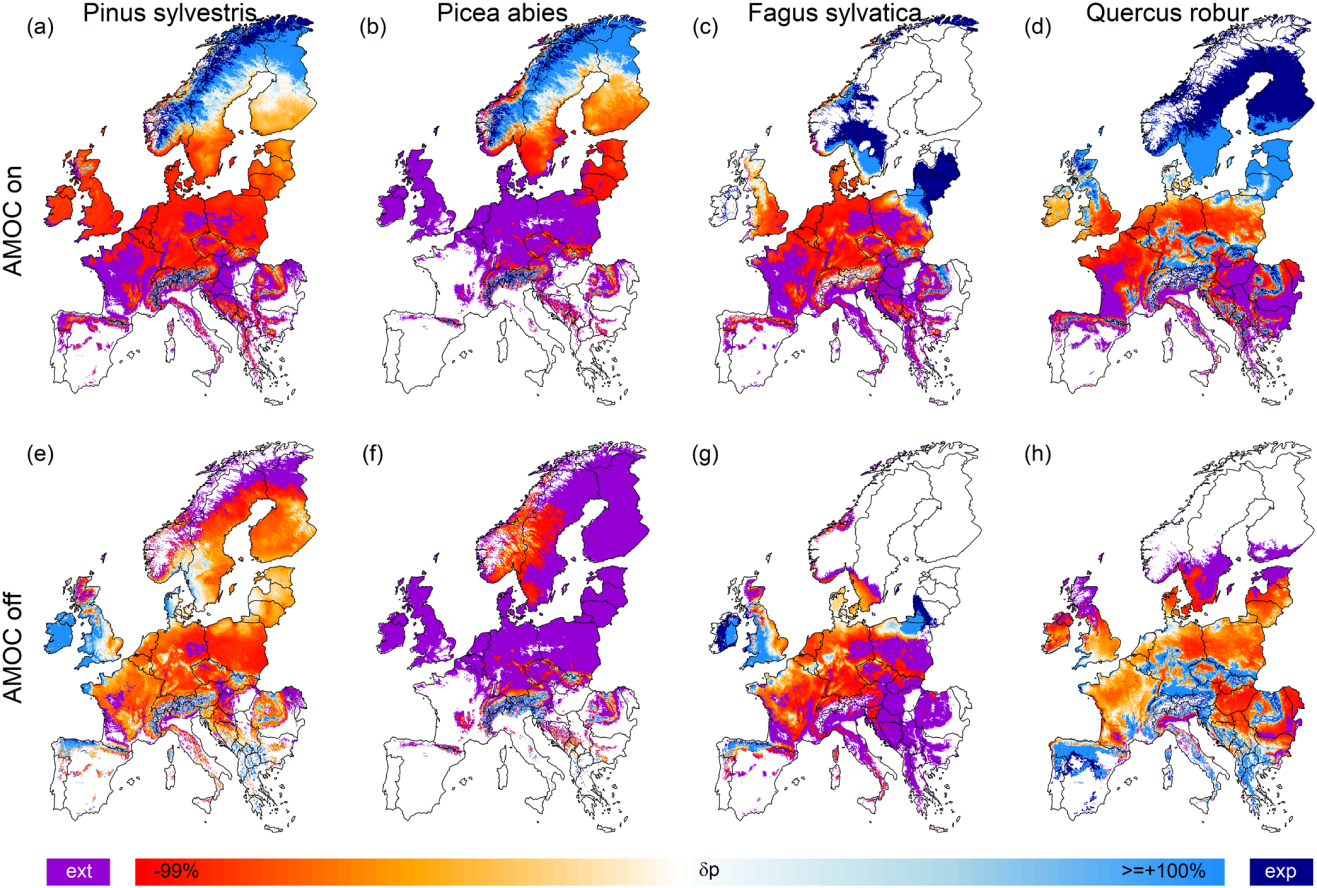
Maps depicting the relative change in occurrence probability *p* (*δp*) between the early historic period and the end of 21st century projection under the SSP2‐4.5 scenario with an active (panels a–d) or inactive (e‐h) AMOC for the four most abundant tree species of Scots pine (a, e), Norway spruce (b, f), European beech (c, g), and Common oak (d, h). *δp* refers to the relative change in *p* with orange‐red colors indicating a decline, and blueish colors indicating an increase. Violet colors indicate a local species extinction (‘ext’) while dark blue colors indicate an expansion (‘exp’) of a given species into the corresponding grid cell. Map lines delineate study areas and do not necessarily depict accepted national boundaries.

**FIGURE 2:**
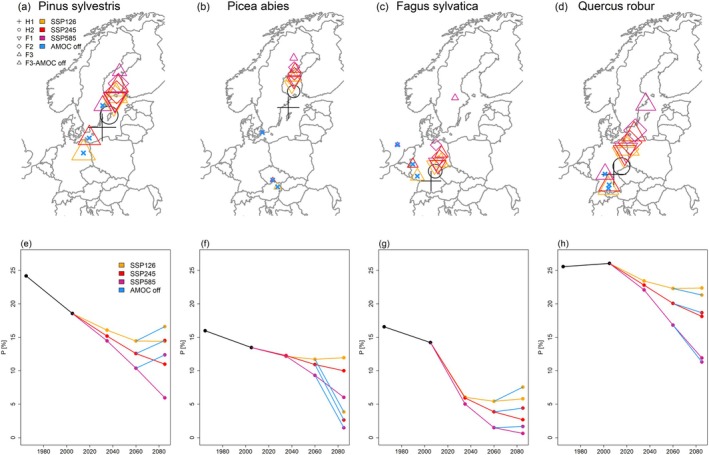
Maps depicting the weighted distributional center (maps in panels a–d) and the mean occurrence probability *p* (e‐h) for Scots pine (a, e), Norway spruce (b, f), European beech (c, g), and Common oak (d, h) under the different scenarios. H1 and H2 refer to the early and late historic period, respectively. F1, F2, and F3 refer to the early, mid, and late future periods, respectively. Color‐code refers to the three different scenarios, while the combination of a triangle with a blue cross inside in the map indicates the AMOC collapse scenario in the late future period. The size of symbols in panels a–d is scaled according to the scenario‐ and period‐specific spatial extent of a given species' distribution. Map lines delineate study areas and do not necessarily depict accepted national boundaries.

Under SSP2‐4.5 conditions, tree‐species projections for the two contrasting AMOC modes (active vs. inactive) displayed contrasting patterns for Europe's currently most abundant tree species (Figures 1 and 2). Under an active AMOC (Figure 1a–d), pine, spruce, beech, and oak were consistently projected for extinction at their southern distributional margins, a moderate (oak) to strong (pine, spruce, beech) decline in their current distributional center, and an increase and expansion at the northern margins. Consequently, their distributional centers consistently shifted northward (Figure 2a–d) while their mean occurrence probability (*p*) decreased (Figure 2e–h).

Under an AMOC collapse (Figure 1e–h) projected tree‐species distributions in contrast showed a diverse picture for these four species. While pine was projected a partial extinction (15% in AMOC off vs. 21% in AMOC on) and comparably strong decline at its northern distributional margins, its projected *p* at its current center and southwestern margin was comparably higher than in the active AMOC scenario (Figure 1e vs. a). Consequently, its overall distribution was located south‐ and westward, yet with marginal changes in spatial extent and a comparably higher overall mean *p* (Figure 2a,e).

Spruce featured the strongest decline under a collapsing AMOC with a projected extinction in 77% (vs. 43%, AMOC on) of its current distribution and gains only in the Alps (Figure 1f). Compared to the active AMOC scenario, its distributional center was relocated from Scandinavia to Central Europe, its distributional extent strongly reduced, and consequently its mean *p* was less than half compared to the active AMOC scenario (Figure 2b,f).

Beech also featured a remarkable extinction (off vs. on: 42% and 34%) and decline at the northern margins under a collapsing AMOC. Similar to pine, *p* in Central Europe was higher than in the active AMOC scenario, yet with higher gains in maritime Western Europe and an ‘exclave’ in the southern Baltic region compared to continental Southeastern Europe (Figure 1g). In combination, its distribution range shrank and shifted westward, while the overall mean *p* increased slightly in comparison to the active AMOC scenario (Figure 2c,g).

For oak, the AMOC collapse resulted in a more or less complete extinction (off vs. on: 14% and 17%) in Scandinavia and Scotland, while *δ*p in Central Europe was higher than in the active AMOC scenario (Figure 1h vs. d). Interestingly, its distribution center remained more or less similar to historic conditions. Yet, in comparison with the active AMOC scenario, it featured a significant southward relocation and a smaller spatial extent (Figure 2d), despite more or less similar mean *p* (Figure 2h).

### 3.2 Projected Changes in Locally Most Abundant Species (Q2)

**FIGURE 3:**
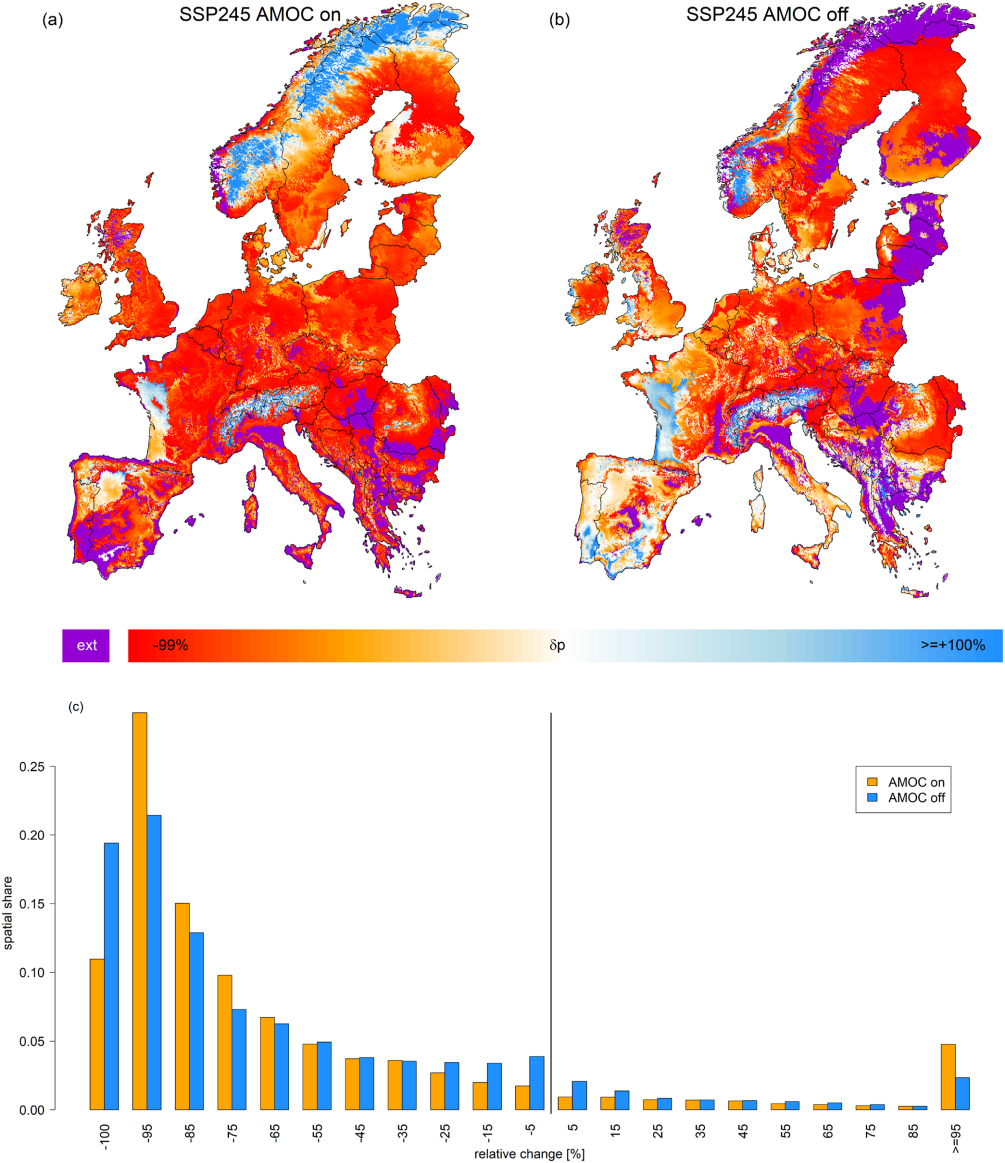
Maps depicting the relative difference of occurrence probability (*δp*) considering all 24 species between the early historic period and the end of 21st century projection under the SSP2‐4.5 scenario with an active (a) or inactive (b) AMOC. In contrast to Figure 1, these maps in each grid cell represent the species that featured the highest occurrence probability during the early historic period of 1951–1980. Panel (c) shows the spatial share of relative change classes, binned into units of 10% steps for the two scenarios. Map lines delineate study areas and do not necessarily depict accepted national boundaries.

When mapping *δp* for the historically most dominant tree species, both AMOC scenarios under SSP2‐4.5 revealed *δp* decline in most regions (Figure 3). Yet, these regions partly differed between the two AMOC scenarios with increasing *p* of the most dominant tree species along the Scandinavian Mountain range under an active AMOC (Figure 3a) and increasing *p* in Southwestern Scandinavia and Southwestern Europe under an inactive AMOC (Figure 3b). The most striking difference between the two AMOC scenarios was represented by local extinction of the respective historically most abundant species in southwestern Europe (AMOC on) which was less pronounced under an inactive AMOC, and local extinctions in Northern Scandinavia and the eastern Baltic (AMOC off) which were not projected for an active AMOC (Figure 3a vs. b). Similarities in extinction between the two AMOC scenarios existed in Northern Italy and Southeastern Europe, indicating regions that are likely to feature a stark change in forest appearance independent of AMOC. Overall, *δp* was more negative under an inactive AMOC, with the area featuring a local extinction (*δp* = −100%) under an inactive AMOC spanning 177% of the extinction area in the active AMOC scenario (Figure 3c).

**FIGURE 4:**
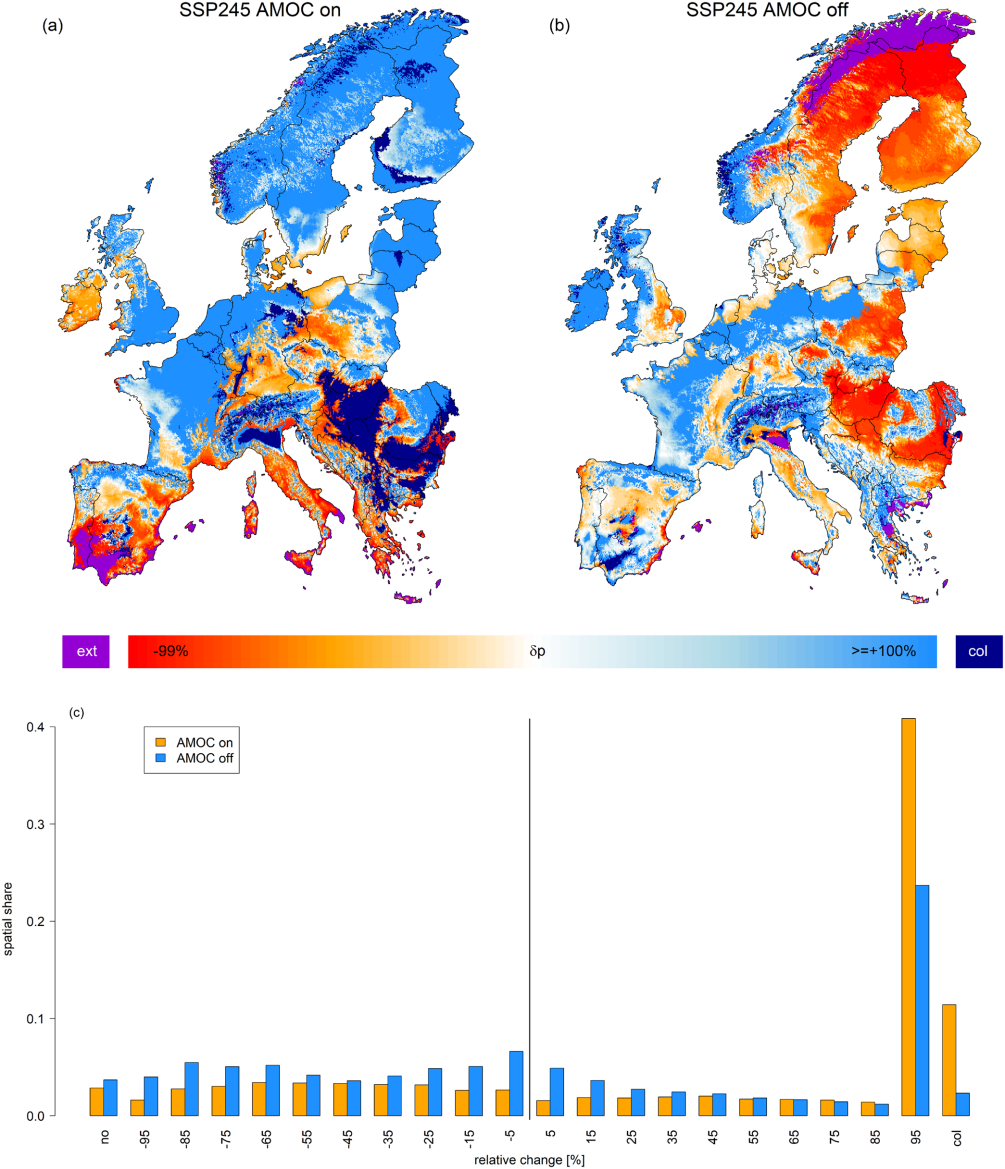
Maps depicting the relative difference of occurrence probability considering all 24 species between the early historic period and the end of 21st century projection under the SSP2‐4.5 scenario with an active (a) or inactive (b) AMOC. In contrast to Figures 1 and 3, these maps in each grid cell represent the species with the highest projected occurrence probability in the late future period (2071–2100). Violet pixels indicate areas where no forest tree‐species was projected occurrence. The dark blue color represents grid‐cells which were newly colonized (‘col’) by the corresponding species. Panel (c) shows the spatial share of relative change classes, binned into units of 10% steps for the two scenarios. Map lines delineate study areas and do not necessarily depict accepted national boundaries.

Mapping *δp* for the species which locally displayed the highest *p* under future conditions, the two AMOC scenarios largely differed. Under an active AMOC, 61% of Europe's area featured a positive *δp* indicating that the species that was projected the highest *p* under future conditions already was present under historic conditions and benefited from climate change (Figure 4a). Only the Mediterranean and some regions in Central and Western Europe indicated that the projected most abundant species featured lower *p* in comparison to today (orange‐red colors in Figure 4a) while none of the considered tree species was projected presence in Southeastern Spain indicating a local disappearance of the considered 24 species (violet color in Figure 4a). Interestingly, in parts of Northern Italy and Southeastern Europe (Pannonian basin) currently absent tree species constituted the future dominant species (dark‐blue color in Figure 4a, total share 11%).

For an inactive AMOC, *δp* of projected dominant species partially indicated a dichotomy between Eastern and Western Europe. While *δp* was mostly positive in Western Europe (light‐blue colors in Figure 4b, total share 42%), it was mostly negative (share: 48%) in Eastern Europe and most of Scandinavia (orange‐red color in Figure 4b). In contrast to an active AMOC, only a few regions (2%) indicated a currently absent species to constitute the projected dominant species (dark‐blue color in Figure 4b), while none of the considered tree species was projected to be present in the Scandinavian Mountain range, suggesting a local forest extinction at boreal high elevation sites (Figure 4b violet color). Overall, *δp* was more negative under an inactive AMOC while the area indicating a novel species to dominate local forests under future conditions only covered 20% of the corresponding area under an active AMOC (Figure 4c).

### 3.3 Substitute Tree Species (Q3)

**FIGURE 5:**
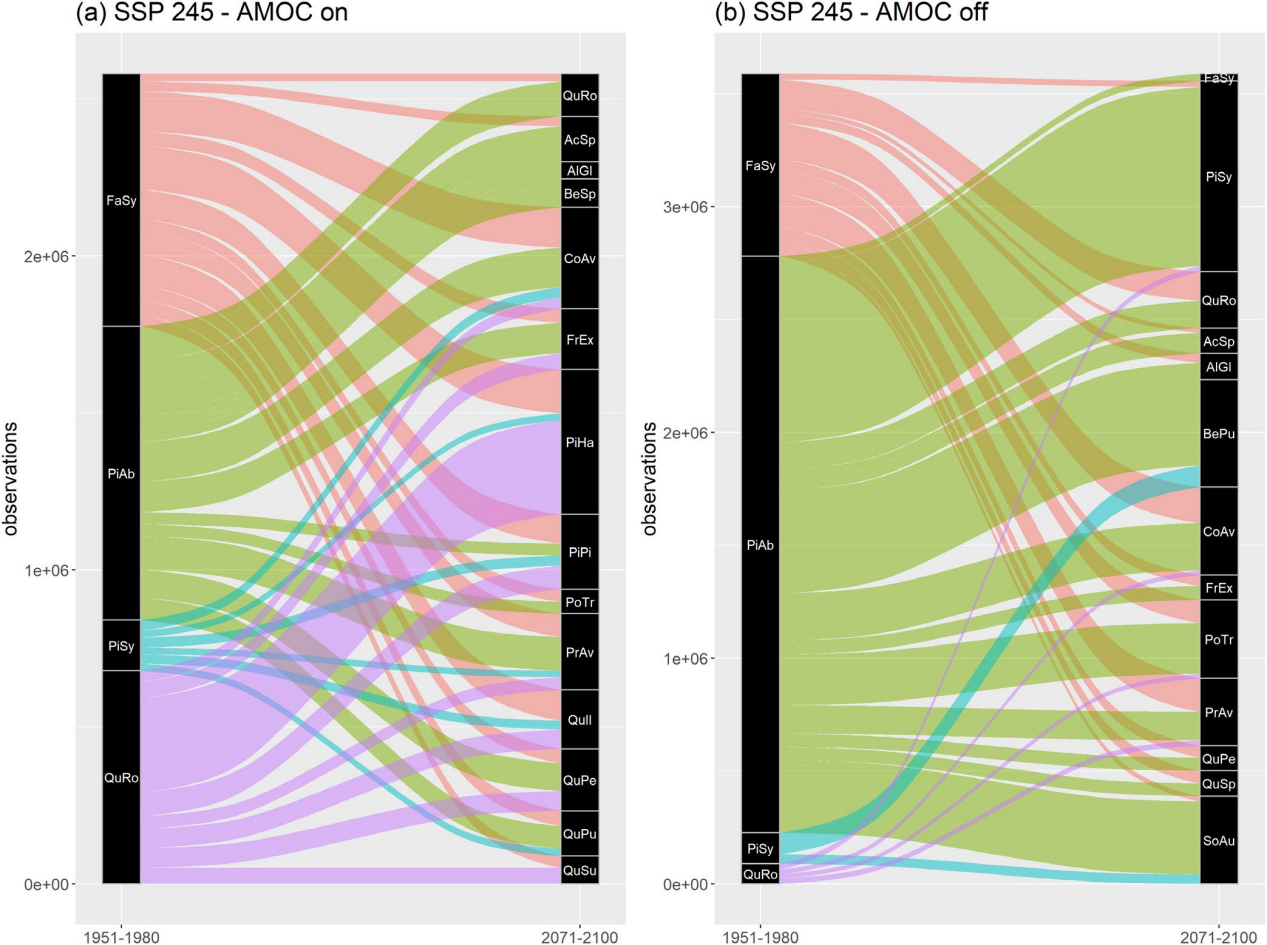
Alluvial plots indicating the species replacing any of the four most abundant tree species in grid cells where those went extinct until the end of the 21st century under the SSP2‐4.5 scenario with an active (a) or inactive (b) AMOC. For the sake of clarity, target species with less than 20,000 observations under future conditions were omitted. For reasons of a better visualization in panel a, *Acer campestre* and *A. pseudoplatanus* were combined to *AcSp*, and *Betula pubescens, B. pendula*, and *Carpinus betulus* were combined to *BeSp*, while in panel b we again classified *AcSp* and combined the mediterranean *Quercus ilex* and *Q. pubescens* to *QuSp*.

Regarding the identification of substitute tree species, the two AMOC scenarios again revealed distinct patterns. Under an active AMOC, the substituting species contained a larger proportion of Mediterranean, drought‐tolerant species such as *Pinus halepensis, Pinus pinaster, Quercus ilex*, and *Quercus pubescens* as well as more temperate species such as *Prunus avium* , *Corylus avellana, Quercus petraea*, and *Fraxinus excelsior* (Figure 5a). In contrast, the inactive AMOC scenario featured a higher share of cold‐tolerant species such as *Pinus sylvestris, Betula pubescens*, *Sorbus aucuparia* , *Corylus avellana* , *Populus tremula* , *Prunus avium* , and *Quercus robur* (Figure 5b). *Corylus avellana* and *Prunus avium* represent species that featured a relatively high share of the portfolio in both scenarios, with each of them replacing any of the four species in more than 200,000 grid‐cells in each scenario.

### 3.4 Projected Changes in Tree‐Species Diversity (Q4)

**FIGURE 6:**
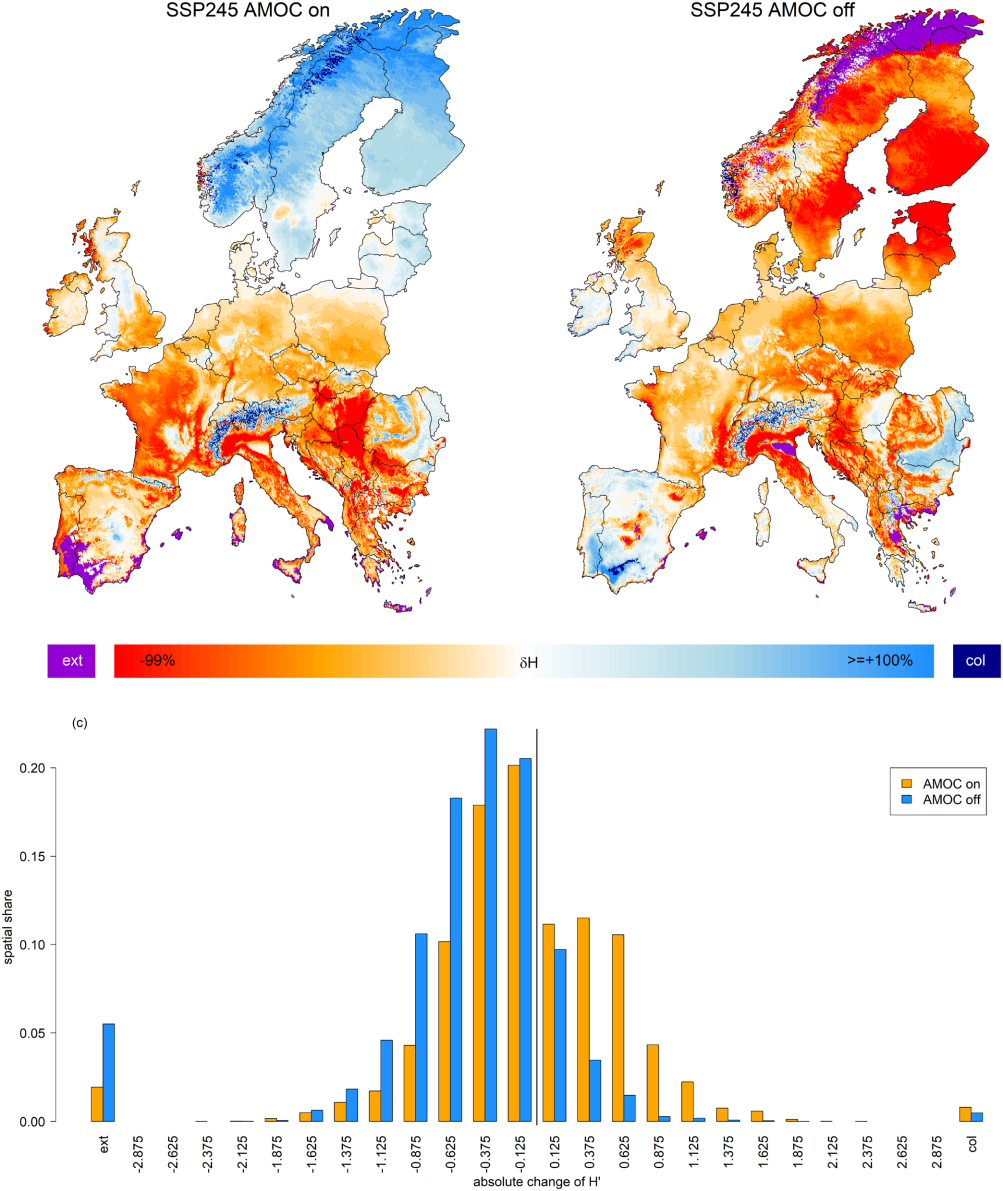
Maps depicting the change in tree‐species diversity (as represented using Shannon's H‐index) for the end of the 21st century under the SSP 2–4.5 scenario with an active (a) or inactive (b) AMOC. Violet color refers to areas where the species under consideration were projected to disappear. Dark blue color refers to pixels which were newly colonized (‘col’) by forest tree species. Panel (c) depicts the spatial share of change classes for the two scenarios. Map lines delineate study areas and do not necessarily depict accepted national boundaries.

Mapping Shannon's H as a measure of potential forest tree‐species diversity, the two AMOC scenarios under SSP2‐4.5 again showed contrasting patterns (Figure 6a vs. b). While these two projections generally agree on a declining diversity in Western, Central, and Southeastern Europe, they largely differ in Northern and Southwestern Europe. That is, while biodiversity is projected to increase in Northern Europe under an active AMOC (light‐blue color in Figure 6a) it is projected a strong decline with local forest extinction (violet color in Figure 6b) under an inactive AMOC. In contrast, the strong decline of diversity and local forest absence on the Iberian Peninsula under an active AMOC (orange‐red and violet color in Figure 6a) is opposed by an increasing diversity and local forest expansion under an inactive AMOC (light‐blue and dark‐blue colors in Figure 6b). Overall, tree‐species richness is projected to decline under both scenarios, yet to a lesser degree under an active AMOC. That is, under an active AMOC, 57% of grid‐cells showed a declining tree‐species diversity, whereas 84% featured declining diversity under an inactive AMOC (Figure 6c).

## 4 Discussion

The general performance of the presented tree‐species projections under an active AMOC is well in line with previous studies (Buras and Menzel 2019; Chakraborty et al. 2021; Dyderski et al. 2018; Koch et al. 2022; Mauri et al. 2022). In particular, the northward (in mountains upward) migration of most tree species into cooler and moister habitats seems a logical consequence due to anticipated warming and drying under climate change. In addition to supporting previous findings at a high spatial resolution of 1 km^2^ and deploying the latest ensemble of climate simulations (CMIP6), our study provides first insights into the effects of an AMOC collapse on European tree‐species distributions.

### 4.1 Effects of an AMOC Collapse on Europe's Forests

In comparison to standard climate‐change projections, a collapsing AMOC would—in dependence of the region and species—partially dampen the effects of climate change due to a local cooling. Yet, the cooling of the North Atlantic would also result in reduced evaporation of water vapor from the ocean and a lower water‐holding capacity of the cooler air, leading to a reduction of annual precipitation sums (Figure S4). As a consequence, pine, beech, and oak would locally suffer less under an AMOC collapse compared to an active AMOC scenario. In contrast, Norway spruce would nevertheless feature a strong decline (Figure 1e–h). In Central Europe, the decline of Norway spruce is likely caused by the drying of the climate, resulting in climate conditions beyond the ‘dry distributional margin’ of spruce which in our climate‐envelope models was determined by the climatic water balance of the driest month (Table S3). Already nowadays, the increasingly drier conditions over the past decades have resulted in Norway spruce suffering from drought‐induced growth decline, bark‐beetle calamities, and dieback (Lévesque et al. 2013; Rehschuh et al. 2017; Schuldt et al. 2020; Spiecker 2000). In the Mediterranean, Common oak benefited from an AMOC collapse as indicated by increasing occurrence probabilities in comparison to historic conditions (Figure 1h). This observation is to be explained by a regional increase in summer precipitation under an AMOC collapse (Figure S4) which represents an important factor for the climate envelope model of Common oak (Table S3).

In Scandinavia, however, all of the four focal species displayed a stark decline, which is to be explained by the strong cooling associated with an AMOC collapse (Figure S4). The only one of the four focal species that would retain a fair share of occurrence probability in Scandinavia is Scots pine (Figure 1e). Norway spruce and Scots pine constitute the economically most important tree species for the Scandinavian timber industry (Felton et al. 2024; Spiecker 2000). A decline of these species, as projected under the AMOC‐collapse scenario, would therefore likely induce major disruptions of Scandinavian forestry, limited lumber deliveries to downstream industries, and significantly higher timber prices (Hanewinkel et al. 2013) in addition to detrimental effects on ecosystem biodiversity.

Apart from the impact on the four focal tree species, an AMOC collapse would cause major changes in the forest's appearance across Europe (Q2). That is, most of the locally abundant tree species would feature a strong decline in occurrence probability. In comparison to an active AMOC scenario, the changes would be less negative in Central Europe and the Mediterranean but worse in Scandinavia and the Baltic States (Figure 3a vs. b).

In many parts of Europe, we observed that locally native tree species would feature an increasing occurrence probability under an active AMOC scenario (Figure 4a). However, under an AMOC collapse, this was only the case for parts of Western Europe, the Alps, and the Mediterranean, while for Eastern Europe and large parts of Scandinavia, the anticipated most suitable tree species featured a much lower occurrence probability than today (Figure 4b). This underscores the detrimental effects an AMOC collapse would have on European forests caused by a cooling‐induced south‐ and downward shift of the thermal tree line (Körner 1998) and a locally drier climate due to reduced precipitation sums.

Concerning alternative tree species that may replace pine, spruce, beech, and oak in regions where they may locally disappear (Q3), the main difference between an active or inactive AMOC refers to a more Mediterranean, drought‐tolerant species portfolio under an active AMOC (Figure 5a) as opposed to a more boreal, cold‐tolerant portfolio under an AMOC collapse (Figure 5b). Interestingly, spruce—which would also disappear widely under an AMOC collapse—would to a remarkable degree be replaced by Scots pine. These results could have important implications for European forest management as the pool of suitable tree species that reliably provide ecosystem services under climate change is further narrowed, compared to species that are promoted for forest management decision‐making by previous modeling studies (Buras and Menzel 2019; Dyderski et al. 2018; Hanewinkel et al. 2013).

For both considered AMOC scenarios, tree‐species diversity was projected to decline in most of Europe (Q4). The observed patterns of species diversity found under an active AMOC largely mirror those of an earlier study (Buras and Menzel 2019), with a declining species diversity in the Mediterranean and Central Europe and an increasing diversity in the Alps and Scandinavia (Figure 6a). In contrast, an AMOC collapse seems to revert the geographical distribution of tree‐species diversity expected in active‐AMOC scenarios, with a much lower diversity in Scandinavia and an increased diversity in the Mediterranean (Figure 6b). This mirrors the observed southward shift of tree‐species distributions due to unfavorable climate conditions in Scandinavia under an AMOC collapse (Figure 2a–d). That is, while the species portfolio of Scandinavian forests is projected to lose some of its species (e.g., spruce), some of the Central European species may find more suitable habitats in the Mediterranean, thereby increasing the local potential tree‐species diversity.

Overall, our projections indicate overly detrimental effects of an AMOC collapse on European tree‐species distributions. While specific species (pine, beech, oak) may locally benefit from an AMOC collapse, some species (e.g., spruce) run at high risk to lose a large part of their current distributional range. While the associated economic losses may be partially outweighed by the selection of alternative tree species with a similar economic importance (e.g., pine replacing spruce in Scandinavia) the associated ecological consequences are likely catastrophic. Moreover, since an AMOC collapse would reduce forest productivity due to cooler temperatures and shorter growing seasons, the carbon sequestration and thus mitigation potential of Scandinavian forests would largely decline. While it remains an open question whether and when an AMOC collapse is to be expected, recent evidence indicates already nowadays a significant reduction of AMOC activity (Caesar et al. 2021) as well as early‐warning indicators that suggest the AMOC to reach a tipping point within the 21st century (Boers 2021; Ditlevsen and Ditlevsen 2023; Michel et al. 2022; Rahmstorf 2024) but see Baker et al. (2025). Given the associated negative impacts of an AMOC collapse on European forests, society should undertake all efforts to lower the risk of an AMOC collapse by a precautionary climate‐change mitigation policy.

### 4.2 Limitations

The presented projections of future tree‐species distributions are affected by several uncertainties. First of all, the selection of the statistical model has a strong impact on the acquired projections. Initially, we also attempted to implement an ensemble of species distribution models (SDM) (e.g., Mauri et al. 2022) as well as a climate‐analogue approach (Buras and Menzel 2019). While the SDM ensemble generally provided satisfying verification results, the application of these models to future climate conditions indicated only minor changes in the distributions of some species. Amongst others, Norway spruce—which already nowadays is affected by widespread decline—was only predicted marginal losses, despite drastic changes in environmental conditions and thus was projected occurrences in regions featuring a future climate outside the historic climate envelope. In our opinion, this feature indicates the ensemble SDM approach to provide unrealistic projections (see Figure S7). In contrast, the climate‐analogue approach appeared to be less suitable for application to very‐high resolution climate data (1 km^2^) as indicated by a very patchy mosaic of adjacent absences and occurrences, which also appears to be unrealistic given very similar climate in adjacent grid cells. A particular advantage of the eventually utilized climate‐envelope approach is that the resulting projections are constrained to the historic climate envelope which remains consistent over time and consequently does not suffer from unrealistic projections as the SDM ensemble (Figure S7). We therefore eventually opted for the climate‐envelope approach, which is directly related to the probability density functions of underlying climatology and consequently featured consistent climate envelopes over all scenarios and periods. As with the SDM ensemble approach, climate envelopes achieved good to excellent verification skills and projected distributions for the early historic period appear to match actual distributions well (Figure S8). Moreover, projected historic changes appeared to be in line with local observations of tree‐species decline. For instance, the reduction of *p* and partial extinction of Norway spruce in Central Europe matches its observed die‐back over most recent decades (Kohler et al. 2010; Spiecker 2000). Also for pine and beech, which have experienced accelerated decline and die‐back over the last decade (Bigler et al. 2006; Buras et al. 2018; Frei et al. 2022; Rigling et al. 2013; Schuldt et al. 2020), corresponding historic changes in *p* seem to match the observations. We therefore consider our model selection robust and suitable for the desired purpose (but see next paragraph).

In our model development, we used the 30‐year climatology of 17 seasonal climate aggregates. While this approach allows for a good representation of climate envelopes, it suffers from the drawback that single extreme events are not represented. Since particularly extreme events have the potential to cause widespread forest decline and tree dieback (Buras et al. 2020; Patacca et al. 2023; Schuldt et al. 2020) it would be desirable to incorporate a higher temporal resolution of the data driving the projections. This, however, would largely increase the already high computational efforts and additionally require the implementation of a more physiological approach as obtained with mechanistic models (Hickler et al. 2012). However, to more accurately simulate tree‐species distributions using mechanistic models, an appropriate implementation of plant hydraulics is required to more precisely simulate drought impacts that ultimately affect tree‐species distributions (Papastefanou et al. 2024, 2020; Peters et al. 2021). The next generation of mechanistic models that currently are under development thus may provide a refined picture of climate change effects on European tree‐species distributions (Meyer et al. 2024).

Within this context, it is important to stress that the presented occurrence probabilities for the species under consideration do not necessarily translate into measures of productivity or carbon sequestration potential. For instance, while our climate envelopes simulate an increased occurrence probability of European beech in Southern Sweden, a recent study reported no growth enhancement of that species within this region under ongoing climate change (Klesse et al. 2024). Thus, we want to stress that the presented projections only reflect climatic habitat suitability, while other environmental factors (e.g., soil conditions, competition, pathogens) also determine whether a species can successfully establish and how much it can grow. In order to obtain reliable growth projections, mechanistic growth models have to be deployed.

Another source of uncertainty comes from the utilized climate projections. It is well known that CMIP models are affected by model‐specific biases and uncertainties (Knutti and Sedláček 2013). Moreover, climate simulations oftentimes come at a spatial resolution that is unsuited to resolve fine‐grained patterns in tree‐species distributions caused by topography (Buras and Menzel 2019). We took great care to lower these effects by quantile mapping the utilized CMIP6 climate variables to historical climate. Thereby, we achieved an almost perfect match between probability density functions of observed and simulated historic climate (Figure S2) and at the same time resolved the inability to map topographic effects by refining the spatial resolution to 1 km^2^. To further account for uncertainty, we deployed ten different CMIP6 models and evaluated ensemble means. As a measure of model uncertainty, we quantified mean and maximum percentual standard deviation of the ensemble projections and partitioned this uncertainty into contributions from models vs. scenarios. Overall, for the focal scenario of SSP2‐4.5, we found the percentual standard deviation to be in the order of a few percent (maxima in the order of 5%) indicating a generally acceptable level of projection uncertainty (Figure S9). By the end of the 21st century, regional differences of uncertainty occurred with highest percentual standard deviations being observed in Southeastern Europe while intermediate values were observed in Central Europe and Scandinavia. The partitioning of this uncertainty across all model‐scenario combinations revealed that models, scenarios, and residuals on average shared an equivalent contribution, yet with regional differences (Figure S10). That is, uncertainty related to models appeared to be particularly high on the Iberian Peninsula and Southeastern Europe while uncertainty related to scenarios was higher in Central Europe and the Baltic States. The residual error—which relates to the interactions among model and scenario uncertainty—showed particularly high contributions in Southeastern Europe, the Iberian Peninsula, and parts of Scandinavia. While more models certainly would be desirable to more comprehensively capture uncertainty, we constrained our selection to ten models given the availability of all required climate variables at the time the data were acquired (see section 2.1). Nevertheless, by using ten models our study likely captures more than 80% of the overall CMIP6 climate range for regional simulations (McSweeney and Jones 2016). Despite the observed generally acceptable uncertainty, future studies should aim at the implementation of more CMIP6 models to further account for model uncertainty. Yet, this comes at the cost of high computational efforts required for quantile mapping the climatic variables and subsequent analyses.

The temperature and precipitation offset used to generate the presented AMOC collapse scenarios is based on one model only (Jackson et al. 2015). Obviously, it would be desirable to incorporate further offsets related to an AMOC collapse from different earth‐system models to account for additional sources of uncertainty. Since the incorporated offsets, however, match reports from other studies relatively well (Rahmstorf 2006; Rahmstorf and Ganopolski 1999), we believe the applied offsets fairly well represent the climatological effects of an AMOC collapse. Nevertheless, future studies should seek to incorporate additional models for quantifying AMOC collapse effects.

Given the available data and model verification, we simulated distributions of 24 different tree species. In this setting, rare species or species for which only limited forest inventory data exist are underrepresented but may play a relevant role in specific regions under climate change in case their climate niches become more abundant. While this does not affect the presented tree‐species projections, it may play a role in regions where our models project local forest extinction. To overcome this limitation, further training data is, however, needed, and future studies should therefore seek to further extend the NFI data utilized here (Mauri et al. 2022).

### 4.3 Summary and Outlook

Our comparative evaluation of the effects of an AMOC collapse on European tree‐species distributions has revealed contrasting responses compared to the classic climate‐change simulations. While an active AMOC would lead to a northward migration of most tree‐species under climate change, an AMOC collapse would result in an opposite redistribution. Given the drier conditions under an AMOC collapse, specific tree‐species—such as Norway spruce—would suffer an even stronger decline throughout Europe since their northernmost habitats—that remain suitable for colonization under an active AMOC—become unfavorable. As a consequence, tree‐species diversity of European forests would feature a stronger decline under an AMOC collapse compared to an active AMOC. Overall, our simulations suggest the effects of an AMOC collapse to have more severe consequences for European forests compared to an active AMOC.

The fact that the economically most important tree species of Norway spruce was heavily affected by an AMOC collapse (Figure 1f) as well as the devastating effects on tree‐species diversity in Scandinavia and Eastern Europe (Figure 6b) highlights the urgency to mitigate the realization of such a scenario. Since the main source of an AMOC collapse—enhanced freshwater input in the North Atlantic due to accelerated ice‐sheet melting (Rahmstorf 2006; Rahmstorf and Ganopolski 1999) – is caused by climate change, effective climate‐change mitigation is the only means to lower the risk of an AMOC collapse.

While our study provides first insights into the detrimental effects of an AMOC collapse on European tree‐species distributions, it yet suffers from several uncertainties related to the input data and model selection (section 4.2). To overcome these limitations, future studies should seek to extend training data and incorporate more mechanistic models instead of empirical models. In particular, adding more tree species to the simulations will provide a more comprehensive picture of potential alternative tree species that qualify as sufficiently robust to cope with various climate‐change scenarios, thereby informing the forestry sector in terms of increasing forests climate‐change resilience.

## Author Contributions

**Sina Heubel:** conceptualization, data curation, formal analysis, investigation, methodology, writing – review and editing. **Anja Rammig:** funding acquisition, supervision, writing – review and editing. **Allan Buras:** conceptualization, data curation, formal analysis, investigation, methodology, project administration, software, supervision, validation, visualization, writing – original draft.

## Conflicts of Interest

The authors declare no conflicts of interest.

## Supporting information


Appendix S1.

## Data Availability

The data and code that support the findings of this study are openly available in Zenodo at http://doi.org/10.5281/zenodo.15101995. Climatologies were obtained from CHELSA (Climatologies at High‐resolution for Earth's Land Surface Areas) at https://chelsa‐climate.org/ (version 2.1). Historic climate data were obtained from CRU (Climate Research Unit) at https://crudata.uea.ac.uk/cru/data/hrg/ (version 4.07). CMIP6 projections of temperature and precipitation used in this study can be found in Table S1. Pan‐European species observations were obtained from Figshare at https://doi.org/10.6084/m9.figshare.c.5525688 and BWI at https://bwi.info/Download/de/BWI‐Basisdaten/.
