# Supplementary material for: The Forest After Tomorrow: Projecting the Impact of a Collapsing Atlantic Meridional Overturning Circulation on European Tree‐Species Distributions
Source: Glob Chang Biol. 2025 Apr 24;31(4):e70185. doi: 10.1111/gcb.70185 (PMC12019781; doi:10.1111/gcb.70185)
Supplement: Supplementary file 1 — Appendix S1. [file GCB-31-e70185-s001.pdf]

Supplementary information to Heubel, Rammig, and Buras

Appendix A – Methodological details

A.1 Downscaling of gridded CRU climate data using CHELSA

For the downscaling, we used the common overlap period of 1981-2010 and computed corresponding monthly climatology of temperature and precipitation for the CRU data, respectively. Subsequently, we remapped the CRU-data to the CHELSA resolution. Since the oftentimes used delta-method is prone to statistical artifacts, we instead opted for application of the change-factor method and divided the CRU climatology with the corresponding climatology from CHELSA. Applying the change-factor method is particularly important for precipitation, for which the delta-method can result in downscaled negative values, which is meaningless for precipitation data. Subsequently, we divided the remapped (1 km^2), monthly CRU data with the corresponding, month- and variable specific change factor. Thereby, we obtained monthly temperature and precipitation time series for the period 1951-2020 at a spatial resolution of 1 km^2 and representative of topographic effects on temperature and precipitation.

A.2 Quantile mapping of CMIP6 projections

For the quantile mapping, we selected the common overlap period between downscaled CRU data (1 km^2) and historic CMIP6 projections, i.e. 1951-2014. For each model-variable-month combination we computed the corresponding quantiles for both downscaled CRU (see A.1) and CMIP6 data. These two quantile distributions were utilized to generate a look-up table at a precision of 0.1 °C (temperature) or 1 mm (precipitation) which allowed for mapping the CMIP6 projections to the distribution of downscaled CRU data. Since the retrieved CRU quantiles contained gaps (not all 0.1 K/ 1mm steps within the full range of CRU data were represented) and also did not cover the full potential range of future climate conditions, gaps inside and outside the range of CRU observations were linearly inter- or extrapolated. Here, we constrained the mapped CMIP6 projections to a range from – 40°C to + 40°C for temperature,

and from 0 to 3000 mm precipitation for precipitation. Since these extreme values never were reached in any of the quantile mapped CMIP6 projections, we considered this range to suffice in terms of climate-variability representation. This approach provided historic CMIP6 projections, whose distributions perfectly matched those of the corresponding historic variables (an example is provided in Appendix C – Fig. S2). Consequently, the bias that is typically observed in CMIP6 projection data was efficiently accounted for while additionally refining the spatial resolution of the data to 1 km^2.

A.3 Extraction of changes in temperature and precipitation from Jackson et al. (2015)

The extraction of temperature- and precipitation-changes from Jackson et al. (2015) was achieved by georeferencing the images and translating the RGB values into the values provided in the corresponding figure legends. Since the figures' color-classification was rather coarse (steps of 1 °C for SAT or 0.1 for RPA, see Jackson et al. 2015), we bilinearly interpolated the obtained maps to a spatial resolution of 1 km^2, resembling the target resolution of quantile mapped CMIP6 projections (see A2). In addition, since the figures did not include latitudes larger than 70° N, we spatially extrapolated the obtained maps to also include northernmost Scandinavia using a bilinear smoother. Finally, since SAT and RPA maps were only representative of winter (DJF) and summer (JJA), we linearly interpolated the maps to also represent spring (MAM) and autumn (SON) periods and downscaled those temporally to resolve monthly values. While we are aware, that the interpolations undertaken introduce statistical artifacts, we are confident that the resulting errors are within the ballpark of the general statistical uncertainty associated with climate projections and thus serve the purpose to represent the changes in surface temperatures and precipitation that would result from an AMOC collapse. To quantify the representativity of the derived maps regarding the original data, we provide a visual comparison which also contain the overall high correlations ($0.87 \leq r \leq 0.93$) between original and derived data (Appendix C - Fig. S3). Maps depicting SAT and RPA for specific months are shown in Appendix C, Figure S4.

A.4 Climate envelope models

In order to determine the best combination of bioclimatic parameters (section 2.1.4.), we tested all possible combinations of two different possible parameter combinations. While the first combination only combined temperature-related parameters with water-availability related parameters, the second combination also included variables related to continentality. This resulted in 42 possible parameter combinations for two parameters and 168 possible parameter combinations if including the 4 continentality parameters, i.e. in total 210 combinations.

For each of the 25 tree-species under consideration we developed 210 different climate envelope models. This was achieved by extracting the relevant bioclimatic parameters at all species-specific observation points (i.e. coordinates from the merged NFI and BWI data) and estimating the parameter-specific probability density function (PDF). Subsequently, we applied the derived PDF to all grid-cells in Europe, to estimate the tree-species occurrence probability *p* of the species under consideration for any of the two (three) parameters. Finally, the obtained occurrence probabilities *p* were conflated with each other using Fisher's combined probability test (Fisher, 1970) which combines a given number of p-values into one test statistic (equation 1),

$$\chi^2_{2k} = -2\sum_{i=1}^{k} \ln p_i \qquad (1)$$

with p_i referring to the p-value of the *i*-th test, and k denominating the total number of p-values to be combined. This statistic follows the chi-squared distribution from which we eventually derived the corresponding p-value. By doing so, we for each tree species and parameter combination obtained occurrence probabilities for each 1 km² grid cell in Europe.

The selection of the final parameter combination for each species was achieved by maximizing the validation statistics. For validation, we firstly trained the model based on 70 % of species-specific observations and computed the omission error for the remaining 30 % of observations.

Next, we computed the true-skill statistic TSS (2), which is achieved by subtracting a value of one from the sum over sensitivity (equation 3) and specificity (equation 4),

$$TSS = Sens + Spec - 1 \tag{2}$$

$$Sens = \frac{a}{a+c} \tag{3}$$

$$Spec = \frac{d}{b+d} \tag{4}$$

where a and d refer to true positives and negatives, respectively, and b and c refer to false positives and negatives, respectively (Allouche et al., 2006). Given the high spatial resolution of the bioclimatic data (1 km^2) and the plot-based nature of the NFI data, there however is a high chance to inflate commission errors. That is, due to the plot-based nature of the utilized occurrence data, model predictions are likely to predict species occurrence in grid cells for which no species-occurrence data exist, although the climatic properties of that grid cell lie well within the climate envelope of a given species. To overcome this problem, we based our assessment of true and false negatives on the chorological map for a given tree species (Caudullo et al., 2017). That is, only the grid cells lying outside the distributional range of a species according to the chorological maps were treated as true absences which were considered for the computation of commission errors. Finally, since TSS relies on presence-absence data, we had to convert the derived occurrence probabilities into a binary format. Since the selection of a threshold occurrence probability for conversion into a binary format is arbitrary, we applied 100 different thresholds in steps of 1 % and from the derived 100 TSS scores selected the maximum value.

For each species, this procedure was repeated for all 210 possible variable combinations from which the best performing model was identified based on the maximum TSS score. In terms of a qualitative classification of TSS scores, we applied the thresholds introduced by Yang et al. (2022) according to which a TSS score below 0.6 refers to average performance, a TSS between

0.6 and 0.8 indicates good performance, and a TSS above 0.8 indicates excellent performance. Here, we only considered models with good or excellent performance, wherefore the model for *Quercus pyrenaica* was not considered for the tree-species projections. An overview on model validation statistics is shown in Appendix B - table S3. A detailed report on the climate-envelope approach in context of ODMAP (Zurell et al., 2020) is presented in Appendix B – table S4.

Appendix B – Supplementary tables

Table S1: Overview on the ten CMIP6 models used. A reference list is provided at the end of the supplementary material.

**Model**	**Institution**	**Reference**
ACCESS-ESM1-5	CSIRO	Ziehn et al. (2019)
CanESM5	CCCma	Swart et al. (2019a)
CanESM5-CanOE	CCCma	Swart et al. (2019b)
CNRM-ESM2-1	CNRM-CERFACS	Seferian (2018)
INM-CM4-8	INM	Volodin et al. (2019a)
INM-CM5-0	INM	Volodin et al. (2019b)
IPSL-CM6A-LR	IPSL	Boucher et al. (2018)
MIROC-ES2L	MIROC	Hajima et al. (2019)
MPI-ESM1-2-LR	MPI-M	Wieners et al. (2019)
UKESM1-0-LL	MOHC	Tang et al. (2019)

Table S2: Bioclimatic parameters utilized for the calibration and prediction of climate envelopes. All parameters refer to means over the corresponding climate normal period of 30 years. Bold font refers to parameters which eventually were used, see also table S3.

Abbreviation	Detailed description of the parameter
MAT	Mean annual temperature
**TWM**	**Mean monthly temperature of the warmest month**
**TCM**	**Mean monthly temperature of the coldest month**
**TJFM**	**Mean temperature of the period January through March**
TJJA	Mean temperature of the period June through August
**GDD**	**Growing degree days, i.e. sum over all positive, linearly interpolated, daily temperatures**
**MACWB**	**Mean annual climatic water balance**
**CWBMA**	**Mean climatic water balance over the period March through August**
**CWBD**	**Mean monthly climatic water balance (P-PET) of the driest month**
CWBW	Mean monthly climatic water balance (P-PET) of the wettest month
CWBJFM	Climatic water balance of the period January through March
CWBJJA	Climatic water balance of the period June through August
WD	Wet days, i.e. sum over all positive, linearly interpolated, daily climatic water balance values
**TVAR**	**Difference between JJA temperature and MAM temperature**
**TR**	**Range of monthly temperature means**
CWBVAR	Difference between JJA temperature and MAM temperature
CWBR	Range of monthly climatic water balance means

Table S3: Validation statistics of the top-candidate climate envelope model for each of the 24 species under consideration. TSS scores indicating an excellent predictive performance are given in bold. The abbreviations are explained in table S2.

Species	Variable selection	Omission error [%]	TSS
Abies alba	GDD, MACWB, TVAR	0.02	0.74
Acer campestre	GDD, CWBD, TVAR	0.02	0.75
Acer pseudoplatanus	TJFM, MACWB, TVAR	0.01	0.65
Alnus glutinosa	GDD, CWBD	0.01	0.76
Alnus incana	TCM, CWBD	0.02	0.66
Betula pendula	GDD, CWBD	0.02	**0.80**
Betula pubescens	TWM, CWBD	0.02	0.78
Carpinus betulus	TWM, CWBD, TVAR	0.02	0.74
Corylus avellana	TJFM, MACWB	0.01	0.79
Fagus sylvatica	GDD, MACWB, TVAR	0.02	0.73
Fraxinus excelsior	TJFM, CWBD	0.01	**0.82**
Picea abies	GDD, CWBD, TR	0.01	0.79
Pinus halepensis	TJFM, CWBD, TVAR	0.02	0.76
Pinus nigra	TWM, CWBD, TR	0.04	0.67
Pinus pinaster	TJFM, CWBD, TR	0.02	0.71
Pinus sylvestris	GDD, MACWB	0.01	0.72
Populus tremula	TCM, CWBMA	0.02	0.78
Prunus avium	TJFM, CWBMA	0.02	0.65
Quercus ilex	TJFM, CWBD, TR	0.02	0.76
Quercus petraea	TJFM, CWBD	0.02	0.78
Quercus pubescens	TWM, CWBD, TVAR	0.02	0.60
Quercus robur	GDD, CWBMA	0.01	**0.80**
Quercus suber	GDD, CWBD, TR	0.02	**0.83**
Sorbus aucuparia	GDD, CWBMA	0.01	**0.85**

Table S4: Detailed reporting on the climate-envelope approach according to the standard ODMAP protocol (Zurell et al., 2020).

ODMAP element	Contents
**Overview**	
Authorship	• **Authors:** Sina Heubel, Anja Rammig, Allan Buras<br>• **Contact:** allan@buras.eu<br>• **Title:** Projecting the impact of a collapsing Atlantic Meridional Overturning Circulation on European tree-species distributions
Model objective	• **Objective:** Forecast and transfer<br>• **Target outputs:** Projected occurrence probability of European tree species under scenarios representing a collapse of the Atlantic Meridional Overturning Circulation (AMOC)
Focal Taxon	Major European tree species
Location	Europe
Scale of analysis	• **Spatial extent**: -10°, 32°, 35°, 72° (xmin, xmax, ymin, ymax)<br>• **Spatial resolution:** 1 km^2<br>• **Temporal extent:** 1951-2100 (CRU + CMIP6); 2071-2100 (AMOC)<br>• **Temporal resolution:** climate normal period (30years)<br>• **Type of extent boundary:** political
Biodiversity Data	• Observation type: standardized monitoring data<br>• Response data type: presence/absence
Predictors	Climatic
Research objective/Hypotheses	Based on recent scientific findings that indicate a potential AMOC collapse until the end of the 21st century, we assessed the impact of an AMOC collapse on European tree species distributions.
Assumptions	Based on our verification statistics, we assume that:<br>• the selected climatic predictor variables are suitable to project species distributions on continental scale<br>• the 30-year climatology represents the climatic envelopes of the selected tree species.
Algorithms	• **Modelling techniques used:** We apply climate-envelope models - Conflation of species-specific probability density functions of 2-3 climate parameters using Fisher's combined probability test (Fisher, 1970).
Workflow	Data preparation (quantile-mapped downscaling of historical and projected climate data, deriving bioclimatic variables), model calibration and validation of climate envelope models including model evaluation (true-skill statistics), predictions with climate envelope models using CMIP6 and AMOC scenarios
Software	• **Software:** All analyses were conducted using R version 4.3.1 (R Core Team, 2023) with packages terra (Hijmans et al., 2025) and Rcpp (Eddelbuettel and François, 2011).<br>• **Data availability:** http://scenarios.forestmonitoringhub.eu/
**Data**	
Biodiversity data	• **Taxon names:** 24 European tree species (*Abies alba, Acer campestre, Acer pseudoplatanus, Alnus glutinosa, Alnus incana, Betula pendula, Betula pubescens, Carpinus betulus, Corylus*

	avellana, Fagus sylvatica, Fraxinus excelsior, Picea abies, Pinus halepensis, Pinus nigra, Pinus pinaster, Pinus sylvestris, Populus tremula, Prunus avium, Quercus ilex, Quercus petraea, Quercus pubescens, Quercus robur, Quercus suber, Sorbus aucuparia)<br>• **Taxonomic reference system:** We follow the taxonomy of EUFORGEN (European forest genetic resources program).<br>• **Ecological level:** species<br>• **Species data sources:** Tree species presence data were obtained from EU-Trees4F dataset (Mauri et al., 2022; https://doi.org/10.1038/s41597-022-01128-5) and complemented with data from Germany`s national forest inventory (Bundeswaldinventur BWI-2012); https://bwi.info/Download/de/BWI-Basisdaten/) for the three most common tree species (Picea abies, Pinus sylvestris, and Fagus sylvatica).<br>• **Sampling design:** 1) EU-Trees4F: EU-Forest data was complemented with occurrence data from intensive monitoring plots and local studies (ICP Forests 2019; Zajac et al., 2001) to fill in geographical areas which were not as well represented for some species. 2) The national forest inventory in Germany (BWI) uses permanent random samples distributed throughout Germany on a regular grid (4 x 4 km). In each sample plot various forest characteristics are recorded.<br>• **Sample size:** The combined tree data set contained 9,651,945 1x1 km cells with a total number of 604,076 occurrences for all species and only 544,910 occurrences for the eventually selected 26 tree species of which two were discarded.<br>• **Scaling:** We added BWI-data to the compiled NFI data for *Picea abies, Pinus sylvestris, and Fagus sylvatica* to overcome an underrepresentation of the actual occurrences of these most abundant species in Central Europe.<br>• **Data cleaning/filtering:** We only considered species with at least 5,900 (approx. 1 % of all data) occurrences and with chorological maps (Caudullo et al., 2017) available for validation.<br>• **Background data:** We use EUFORGEN`s chorological maps (Caudullo et al., 2017) for validation.
Data partitioning	• **Training data:** We randomly selected 70% of the species occurrence data for model building.<br>• **Validation data:** We selected the remaining 30% of the data for validation of the predictions.
Predictor variables	• **Predictor variables:** 17 bioclimatic parameters were derived from historic and future climate projections of temperature and precipitation. Eventually, 9 of these 17 parameters turned out to provide robust models with good to excellent predictive skills. These parameters refer to: mean monthly temperature of the warmest month (TWM), mean monthly temperature of the coldest month (TCM), mean temperature of the period January through March TJFM), growing degree days (GDD), mean annual climatic water balance (MACWB), mean climatic water balance over the period March through August (CWBMA), mean climatic water balance of the driest month

	(CWBD), standard deviation of monthly temperature (TVAR), range of monthly temperature means (TR).<br>• **Data sources:** Temperature and precipitation data was obtained from ten models in the CMIP6 ensemble (https://aims2.llnl.gov/search/cmip6/) and complemented with data from Jackson et al. (2015) for the scenario representing an AMOC collapse. A complete overview of data sources can be found in the original publication and supplementary materials.<br>• **Spatial extent:** -180, 180, -90, 90 (xmin, xmax, ymin, ymax)<br>• **Spatial resolution:** >0.5° (the spatial resolution of the CMIP6 data depends on the individual climate model)<br>• **Coordinate reference system**: EPSG:4326 (WGS 84)<br>• **Temporal extent:** Climate data was obtained for two historic climate normal periods (1951-1980, 1991-2020) and three future periods (2021-2050, 2046-2075, 2071-2100).<br>• **Temporal resolution:** The temporal resolution of the climate data corresponds to a 30-year climate normal period.<br>• **Data processing:**<br>1) CMIP6 projections were downscaled to 1km spatial resolution applying a quantile mapping approach (Ekström et al., 2015) with statistically downscaled, historical reference data. Details of this downscaling are provided in Appendix A.2 of the supplementary information.<br>2) We calculated 9 bioclimatic predictors (mentioned above) that are related to seasonal integrations of temperature and climatic water balance. All derived parameters were averaged for each grid cell over two historic climate normal periods (1951-1980, 1991-2020) and three future periods (2021-2050, 2046-2075, 2071-2100) for the CMIP6 scenarios and for the period 2071-2100 for the AMOC collapse scenarios. An overview is presented in table S2 of the Appendix B of the supplementary information.
Transfer data	• **Data sources:**<br>1) We used Climate Research Unit (CRU) historical reference data (CRU TS version 4.07; Harris et al., 2020) which were statistically downscaled using CHELSA climatologies (CHELSA version 2.1, Karger et al., 2023)<br>2) We extracted information from figures 4 and 7 presented in Jackson et al. (2015) to obtain projections of temperature and precipitation which resemble an AMOC collapse<br>• **Spatial extent:** CRU and CHELSA: -180, 180, -90, 90 (xmin, xmax, ymin, ymax); AMOC: -10, 32, 35, 72 (xmin, xmax, ymin, ymax)<br>• **Spatial resolution:** 0.5° (CRU), 1 km^2 (CHELSA), 60 km (AMOC)<br>• **Temporal extent:** 1901-2023 (CRU), 1981-2010 (CHELSA), 100 simulation years (AMOC)<br>• **Data processing:**<br>3) We used the CHELSA climatologies to statistically downscale the CRU gridded temperature and precipitation

	data. Details of this downscaling are provided in Appendix A.1 of the supplementary information.<br>4) We superimposed the data extracted from figures 4 and 7 presented in Jackson et al. (2015) on the quantile mapped CMIP6 temperature and precipitation projections. Details are provided in Appendix A.3 of the supplementary information.<br>• **Quantification of novelty:** No previous study has assessed the impact of AMOC collapse on tree-species distributions
**Model**	
Variable pre-selection	In order to determine the best combination of bioclimatic parameters, we tested all feasible combinations of different possible parameter combinations (either 2 or 3 variables in combination). The selection of the final parameter combination for each species was achieved by maximizing the validation statistics (TSS).
Model selection	We used climate envelope models to project future tree species distributions, which are directly related to the probability density functions of underlying climatologies, based on satisfying verification statistics and projected changes in line with actual distributions. Initially, we also attempted to implement an ensemble of species distribution models (SDM) (e.g. Mauri et al., 2022) as well as a climate-analogue approach (Buras and Menzel, 2019). While the SDM ensemble generally provided satisfying verification results, application of these models to future climate conditions indicated only minor changes in the distributions of some species. In contrast, the climate-analogue approach appeared to be less suitable for application to very-high resolution climate data (1 km^2) as indicated by a very patchy mosaic of adjacent absences and occurrences. Justifications on the selection of climate envelope models are presented in Appendix A.4 and Appendix C of the supplementary items of the published article (namely Figs. S7).
Threshold Selection	No threshold was selected: We mapped actual probabilities of occurrence.
**Assessment**	
Performance statistics	• **Performance statistics estimated on training data:** Model performance was assessed based on the average true skill statistic (TSS)<br>• **Performance statistics estimated on validation data:** TSS
Plausibility checks	• **Expert judgements:** Model plausibility was checked by map display and agreement with chorological maps of current species distributions as well as related peer-reviewed scientific studies.
**Prediction**	
Prediction output	• **Prediction unit**: For our further analyses, we used predictions of future occurrence probability per analyzed species and grid cell expressed on a continuous scale (0-100).<br>• **Post-processing:** From the projected occurrence probabilities we calculated relative changes in occurrence probability between the early historic period (1951-1980) and the future projections; we computed a weighted geometric mean across

	coordinates representative of a species' distribution center under the various scenarios to reflect changes in the actual distribution of a species; we computed the mean, area-weighted occurrence probability over all grid cells in which the species was projected to occur; and we calculated Shannon`s biodiversity index H' and corresponding changes in H' between the early historic period (1951-1980) and the future projections.
Uncertainty quantification	• **Algorithmic uncertainty:** Uncertainty of predictions was quantified by computing species-specific, proportional standard deviations of projected occurrence probability in relation to the mean projected occurrence probability over all ten models. Moreover, we assigned the identified uncertainty to models and scenarios by computing pixel-specific ANOVAs and deriving the relative share of the sum of squares related to models, scenarios and the residual error term (Diniz-Filho et al., 2009).<br>• **Uncertainty in input data:** Underrepresentation of most common species in compiled European NFI data: We combined the compiled European national forest inventory data with BWI-data (national forest inventory data of Germany) which resulted in a stronger representation of lowland occurrences of *Picea abies, Pinus sylvestris*, and *Fagus sylvatica* and hence a more robust representation of the actual current distribution of these species.<br>• **Scenario uncertainty (climate models, storylines):**<br>1) Uncertainty in climate models: We applied an ensemble approach averaging over ten different CMIP6 models.<br>2) Uncertainty in emission scenarios: We used three emission scenarios SSP-1 2.6, SSP-2 4.5, SSP-5 8.5.<br>• **Visualization/treatment of novel environments:** We represent changes in species` occurrence probability in corresponding maps. These can be visualized and downloaded for all analyzed tree species in an interactive shiny-app: http://scenarios.forestmonitoringhub.eu .

Appendix C – Supplementary display items

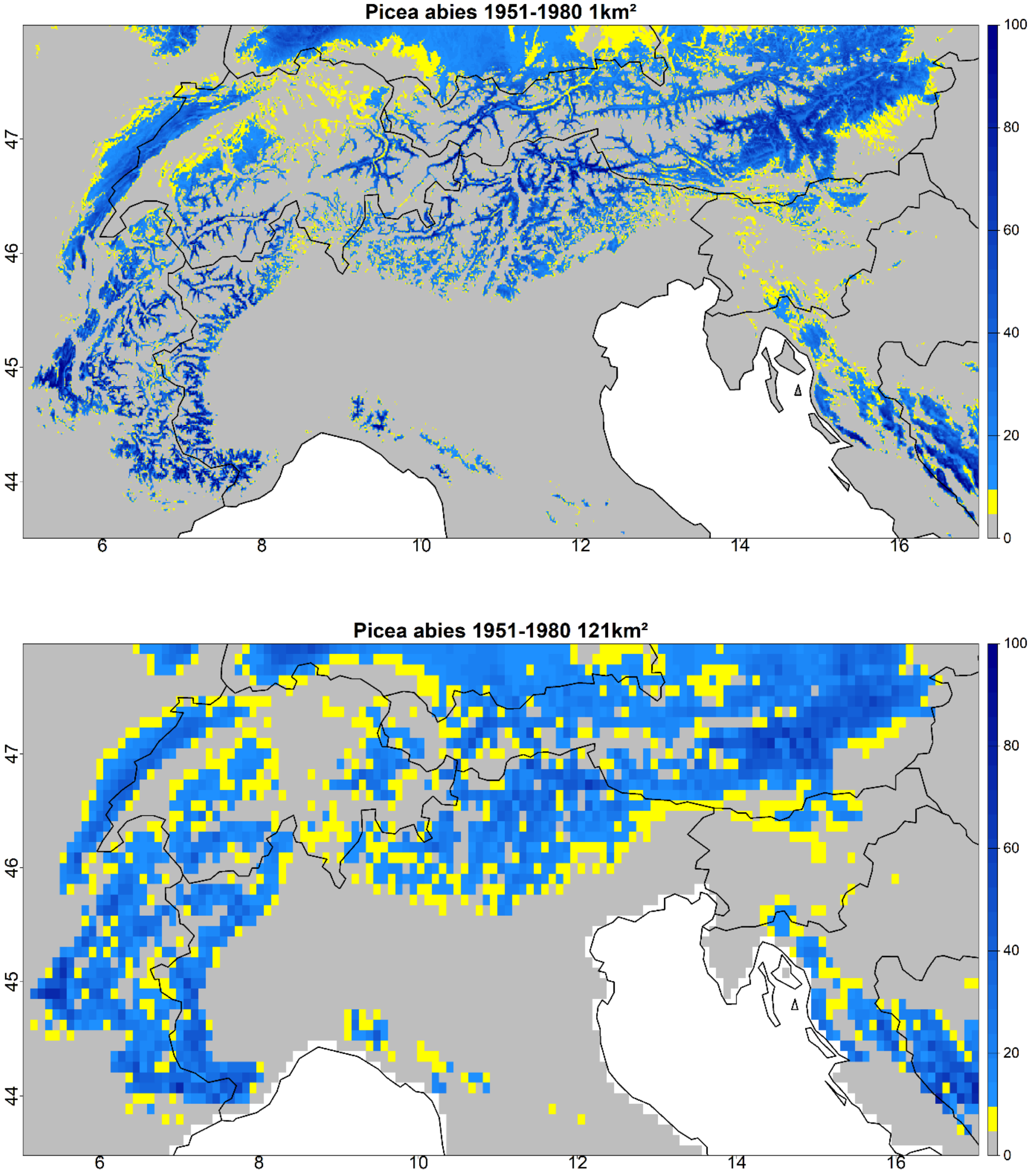

Fig. S1: Close-up of the projected distribution of Norway spruce in the early historic period, exemplifying the high level of detail obtained with the spatial resolution of 1 km² (upper panel). In comparison (lower panel), a lower resolution of 11 km² would not resolve the fine-grained topographic effects of inner-alpine valleys. Map lines delineate study areas and do not necessarily depict accepted national boundaries.

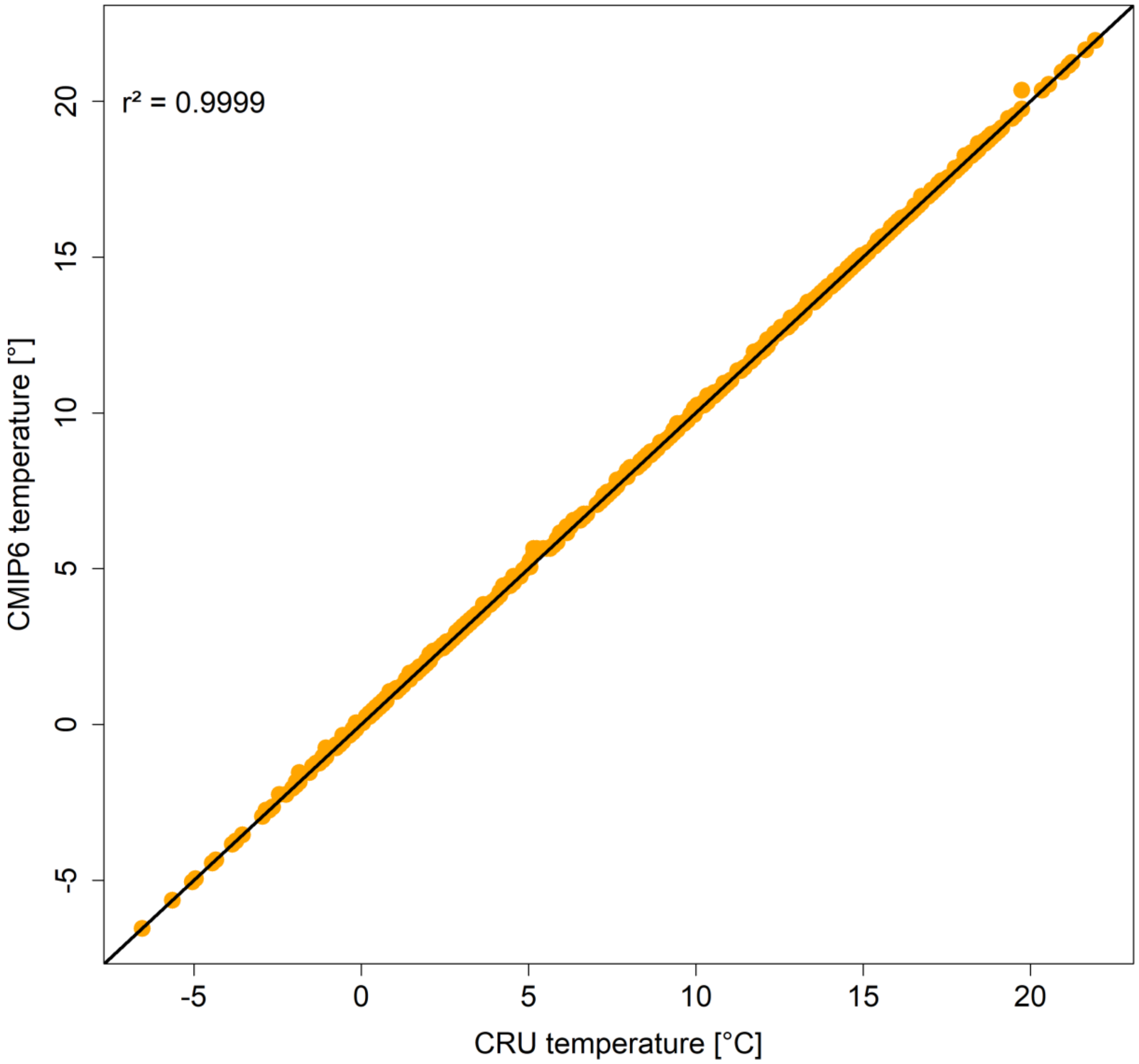

Figure S2: Scatterplot depicting the excellent match of quantile-mapped temperatures between source (CRU-1km²) and target (CMIP6). R^2 refers to the explained variance of a linear regression between the two variables ($p < 0.001$). The black line demarcates a regression with slope one and intersect zero.

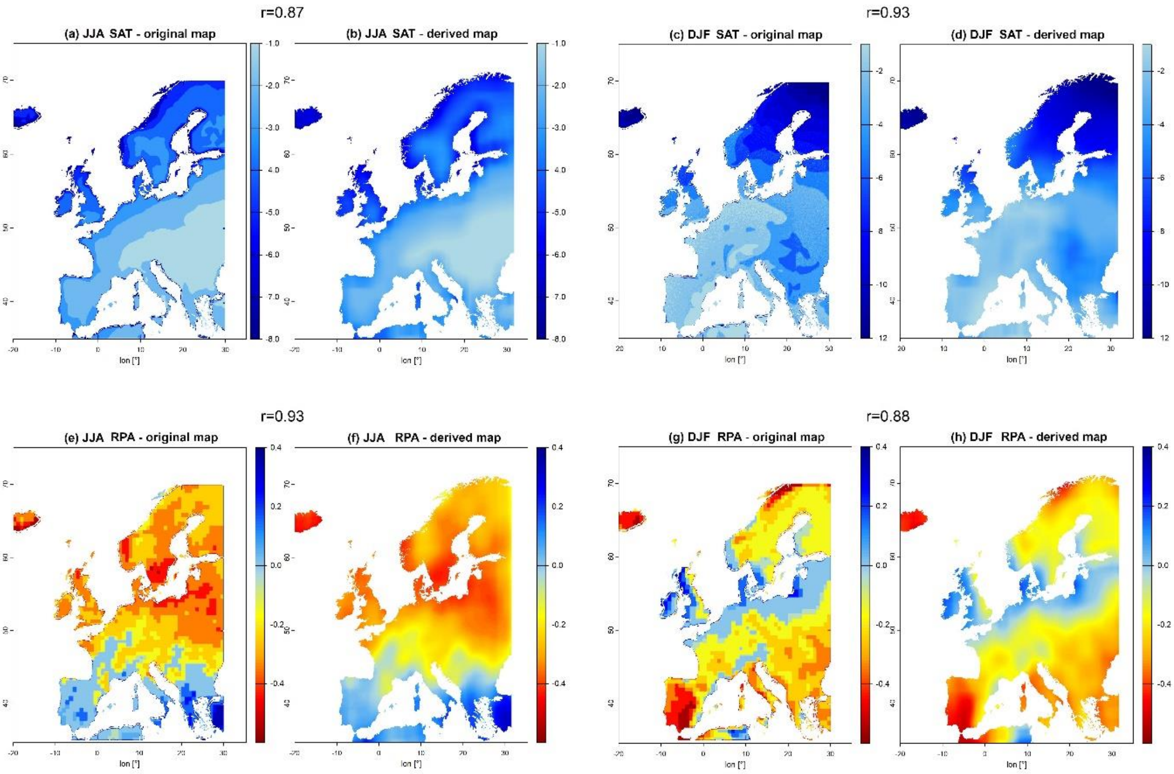

Fig. S3: Maps depicting the agreement between the original data presented in Jackson et al. (2015) and the maps derived from the original data for JJA SAT (panels a vs. b), DJF SAT (c vs. d), JJA RPA (e vs. f), and DJF RPA (g vs. h). Correlation scores between original and derived data are presented above each of the comparisons. All correlations were highly significant ($p < 0.001$).

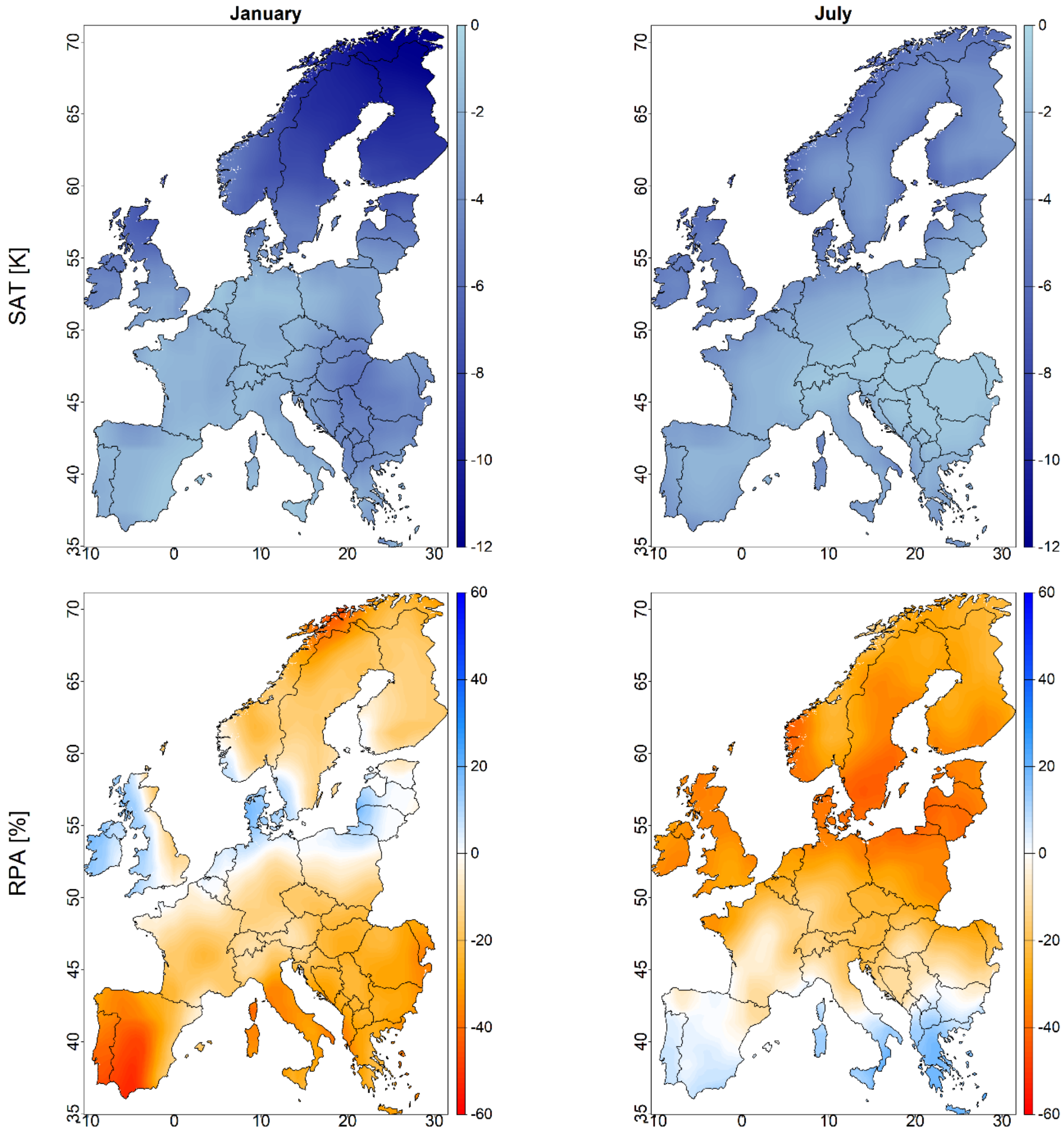

Fig. S4: Maps depicting the surface temperature anomaly (SAT, top panels) and relative precipitation anomaly (RPA, bottom panels) for January (left panels) and July (right panels) representative of an AMOC collapse as derived from Jackson et al. (2015). For details on how the data were generated, see main text. Map lines delineate study areas and do not necessarily depict accepted national boundaries.

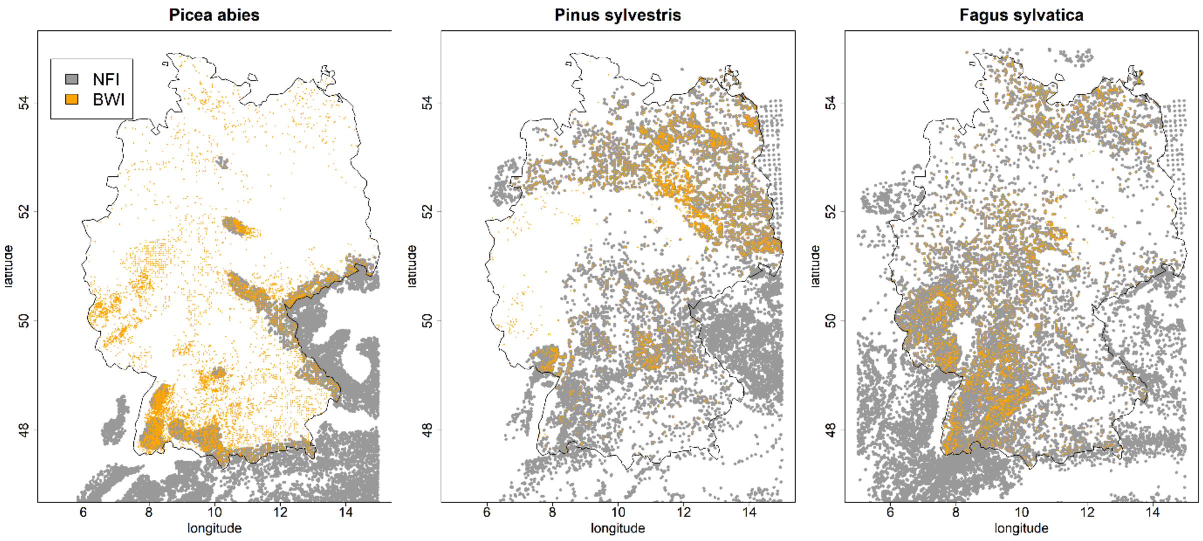

Fig. S5: Maps depicting the overlap between NFI data (Mauri et al., 2022) and BWI data for the area around Germany. In particular for Norway spruce (*Picea abies*) the BWI data provide a substantial complementation to the NFI data. Map lines delineate study areas and do not necessarily depict accepted national boundaries.

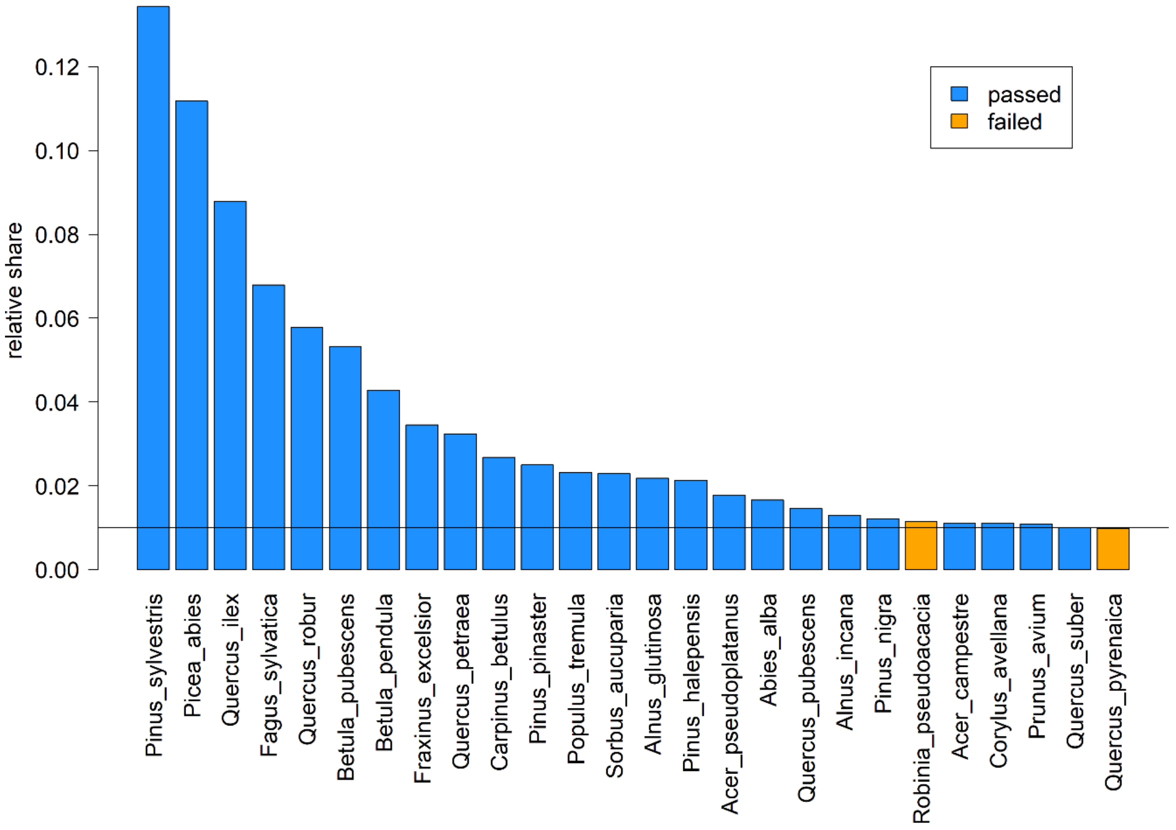

Fig. S6: Barplot depicting the relative share of species' contributions to the combined national forest inventory data used as training data for the calibration of climate species-specific envelopes. The term 'passed' (failed) refers to a(n) (un-) successful climate-envelope calibration.

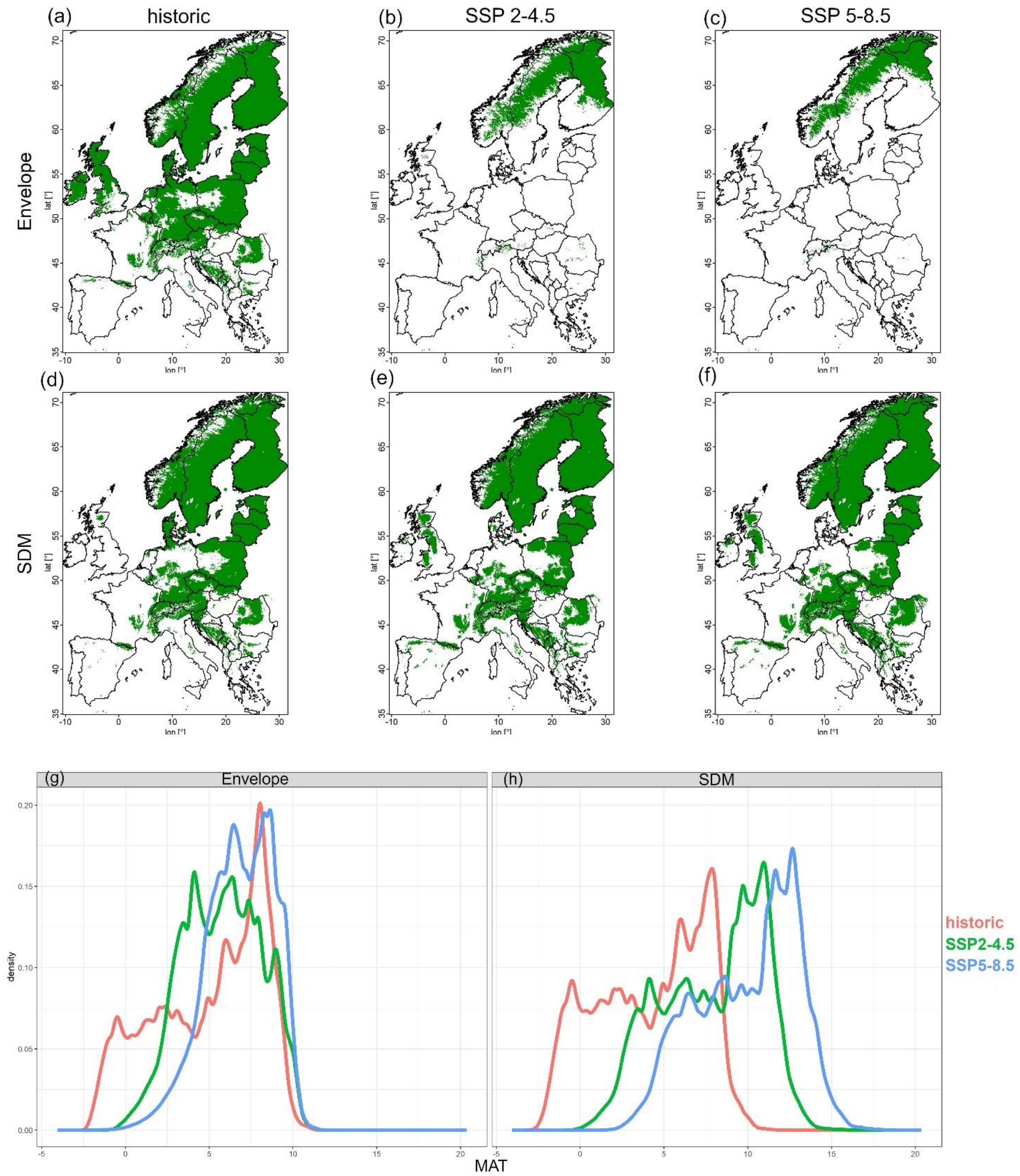

Fig. S7: Comparison between projected occurrences of *Picea abies* under different future scenarios for climate envelope models ('Envelope', panels a-c) and SDM model ensembles ('SDM', panels d-f). Maps indicate regions with projected occurrence probability larger than 10% while the density charts below (panels g-h) indicate the projected mean annual temperatures (MAT) of these regions. While the climate envelope model projections are constrained to the historic MAT envelope (g), MAT of projected SDM ensemble occurrences was above the historic thermal maximum in up to 20 % of pixels for SSP5-8.5 (h). Map lines delineate study areas and do not necessarily depict accepted national boundaries.

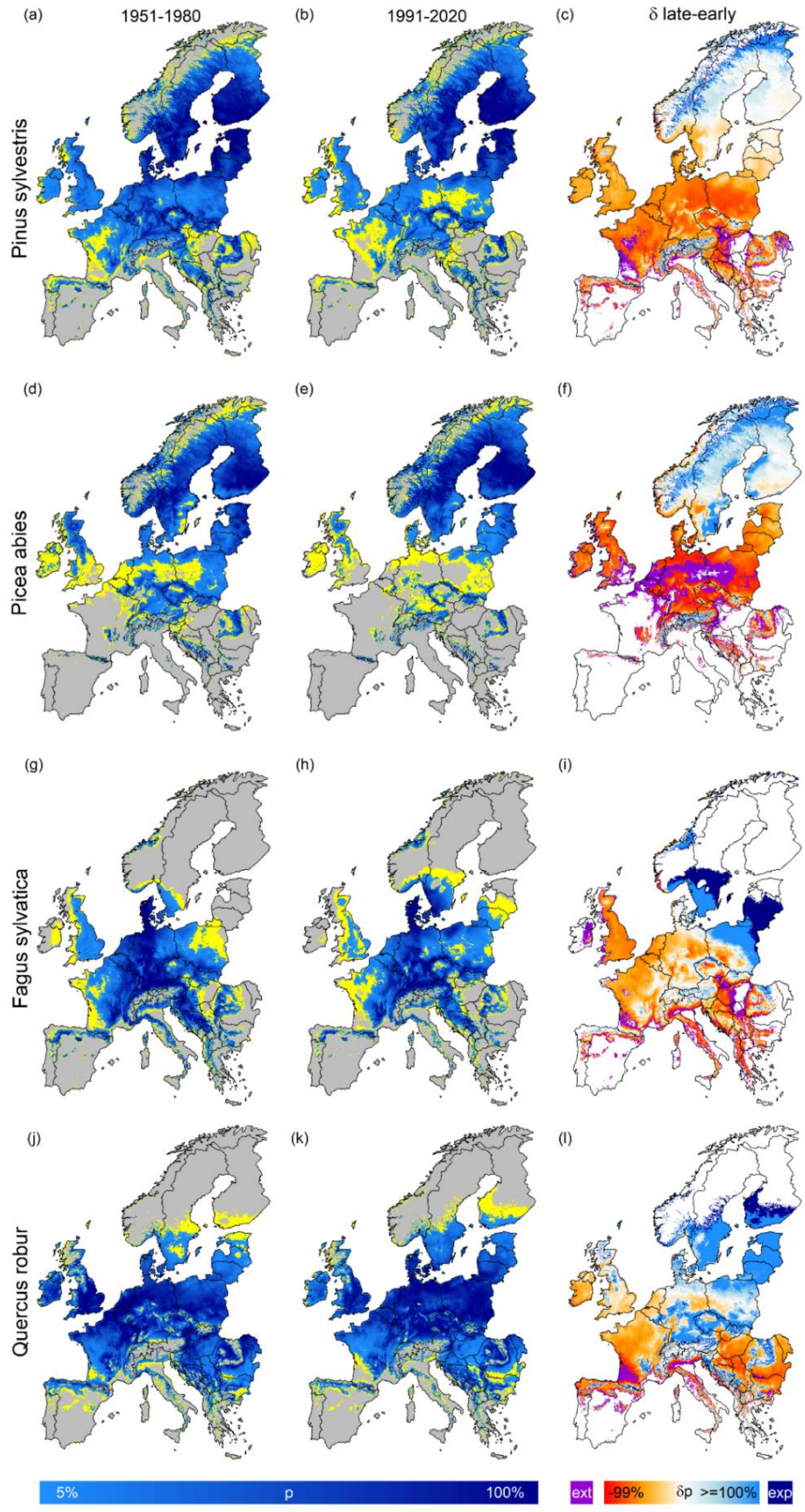

Fig. S8: Tree-species projections for the four most common European tree-species of Scots pine (a-c), Norway spruce (d-f), European beech (g-i) and Common oak (j-l) for the early historic period (a, d, g, j), the late historic period (b, e, h, k), as well as the relative difference between the projected occurrence probabilities (c, f, i, l). Map lines delineate study areas and do not necessarily depict accepted national boundaries.

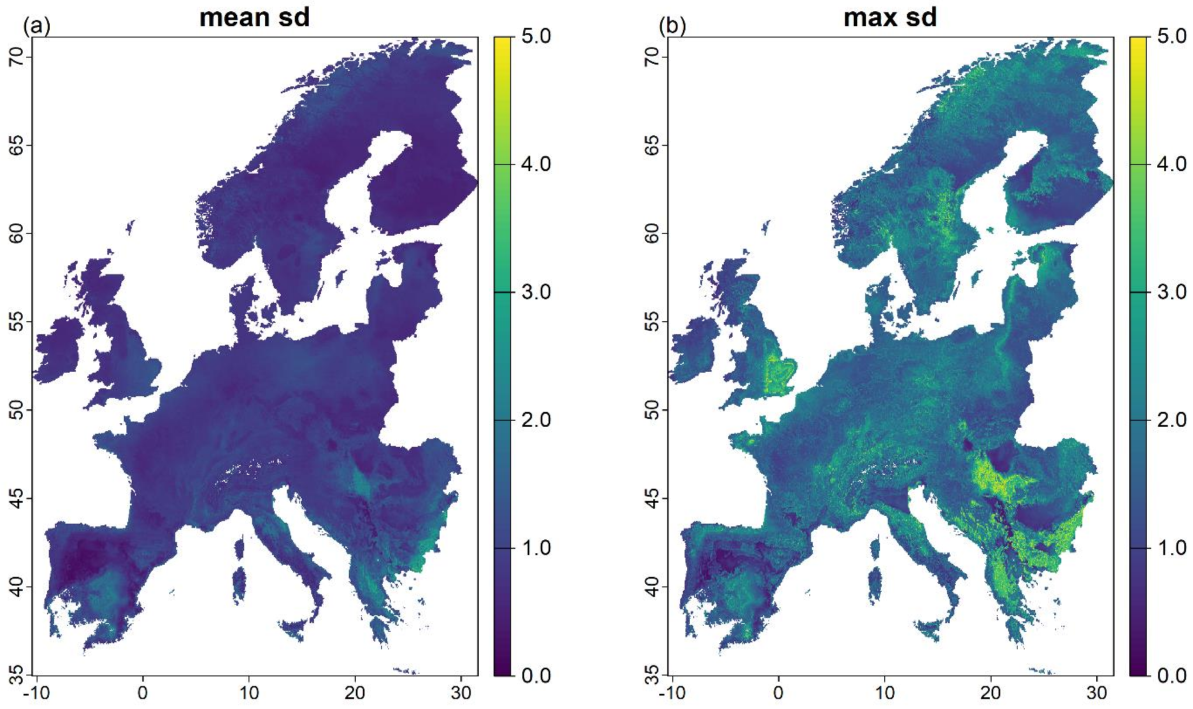

Fig. S9: Mean (a) and maximum (b) percentual standard deviations of projected occurrence probabilities over all species and across the model ensemble. Values are given in % of the mean occurrence probability *p*.

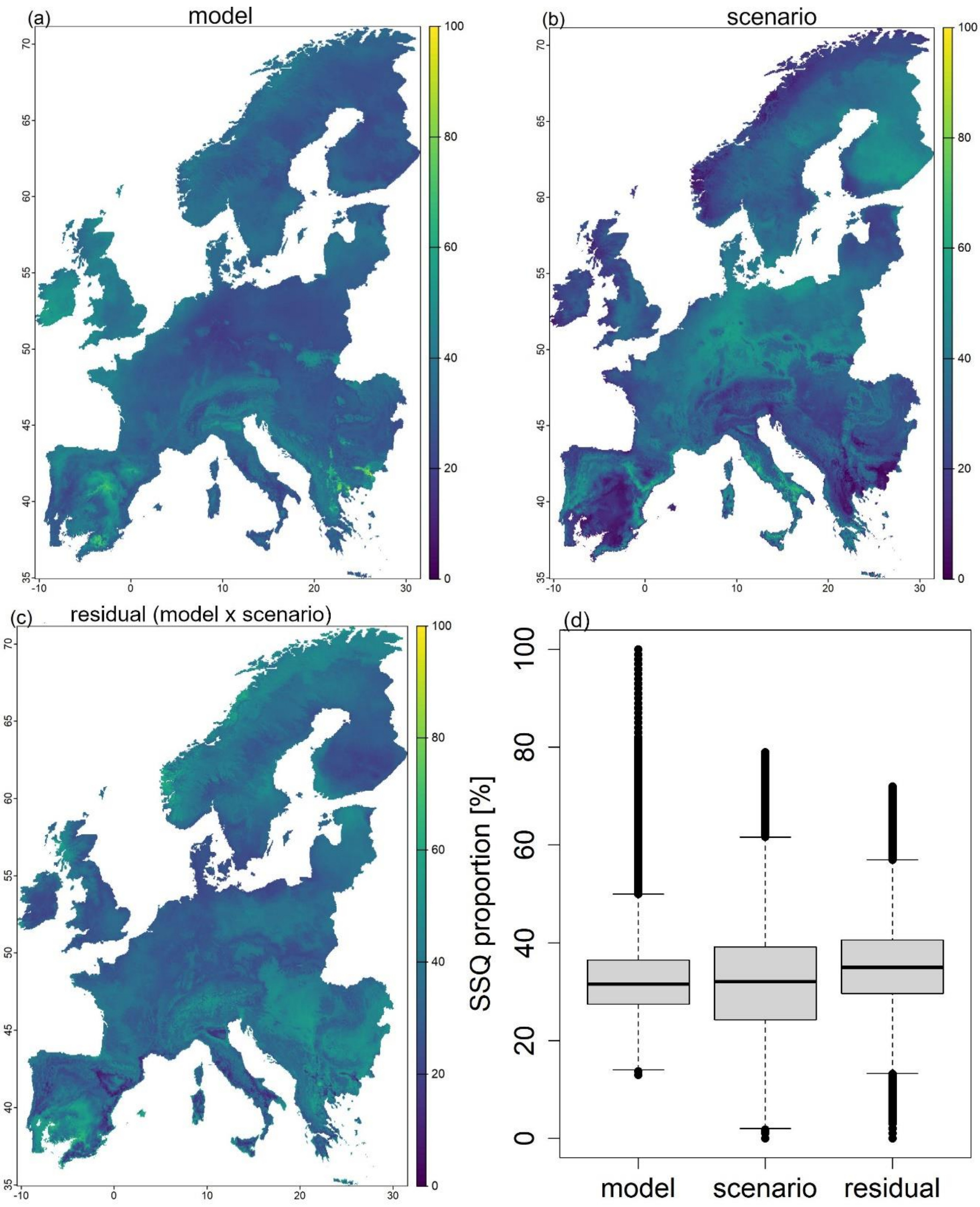

Fig. S10: Partitioning of projection uncertainty into contributions from models (a), scenarios (b), and the residual error (c) which represents interactions among both. Panel (d) provides a direct comparison of values among the three components.

Supplementary references:

Allouche, O., Tsoar, A., Kadmon, R., 2006. Assessing the accuracy of species distribution models: prevalence, kappa and the true skill statistic (TSS). Journal of Applied Ecology 43, 1223–1232. https://doi.org/10.1111/j.1365-2664.2006.01214.x

Boucher, Olivier et al. (2018). IPSL IPSL-CM6A-LR model output prepared for CMIP6 CMIP. Retrieved: August 2022. Earth System Grid Federation. doi:https://doi.org/10.22033/ESGF/CMIP6.1534

Buras, A., Menzel, A., 2019. Projecting Tree Species Composition Changes of European Forests for 2061–2090 Under RCP 4.5 and RCP 8.5 Scenarios. Front. Plant Sci. 9. https://doi.org/10.3389/fpls.2018.01986

Caudullo, G., Welk, E., San-Miguel-Ayanz, J., 2017. Chorological maps for the main European woody species. Data in Brief 12, 662–666. https://doi.org/10.1016/j.dib.2017.05.007

Diniz-Filho, J. A. F., Mauricio Bini, L., Fernando Rangel, T., Loyola, R. D., Hof, C., Nogués-Bravo, D., & Araújo, M. B. (2009). Partitioning and mapping uncertainties in ensembles of forecasts of species turnover under climate change. Ecography, 32(6), 897-906.

Eddelbuettel, D., François, R., 2011. Rcpp: Seamless R and C++ integration. Journal of statistical software 40, 1–18.

Ekström, M., Grose, M.R., Whetton, P.H., 2015. An appraisal of downscaling methods used in climate change research. WIREs Climate Change 6, 301–319. https://doi.org/10.1002/wcc.339

Fisher, R.A., 1970. Statistical methods for research workers, in: Breakthroughs in Statistics: Methodology and Distribution. Springer, pp. 66–70.

Hajima, Tomohiro et al. (2019). MIROC MIROC-ES2L model output prepared for CMIP6 CMIP. Retrieved: August 2022. Earth System Grid Federation. doi:https://doi.org/10.22033/ESGF/CMIP6.902

Harris, I., Osborn, T.J., Jones, P., Lister, D., 2020. Version 4 of the CRU TS monthly high-resolution gridded multivariate climate dataset. Sci Data 7, 109. https://doi.org/10.1038/s41597-020-0453-3

Hijmans R (2025). terra: Spatial Data Analysis. R package version 1.8-29, https://github.com/rspatial/terra.

ICP Forests. International Co-operative Programme on Assessment and Monitoring of Air Pollution Effects on Forests. http://icp-forests.net/ (2019).

Jackson, L.C., Kahana, R., Graham, T., Ringer, M.A., Woollings, T., Mecking, J.V., Wood, R.A., 2015. Global and European climate impacts of a slowdown of the AMOC in a high resolution GCM. Clim Dyn 45, 3299–3316. https://doi.org/10.1007/s00382-015-2540-2

Karger, D.N., Lange, S., Hari, C., Reyer, C.P.O., Conrad, O., Zimmermann, N.E., Frieler, K., 2023. CHELSA-W5E5: daily 1 km meteorological forcing data for climate impact studies. Earth System Science Data 15, 2445–2464. https://doi.org/10.5194/essd-15-2445-2023

Mauri, A., Girardello, M., Strona, G., Beck, P.S.A., Forzieri, G., Caudullo, G., Manca, F., Cescatti, A., 2022. EU-Trees4F, a dataset on the future distribution of European tree species. Sci Data 9, 37. https://doi.org/10.1038/s41597-022-01128-5R Core Team

Seferian, Roland (2018). CNRM-CERFACS CNRM-ESM2-1 model output prepared for CMIP6 CMIP. Retrieved: August 2022. Earth System Grid Federation. doi:https://doi.org/10.22033/ESGF/CMIP6.1391

Swart, Neil Cameron et al. (2019a). CCCma CanESM5 model output prepared for CMIP6 CMIP. Retrieved: August 2022. Earth System Grid Federation. doi:https://doi.org/10.22033/ESGF/CMIP6.1303

Swart, Neil Cameron et al. (2019b). CCCma CanESM5-CanOE model output prepared for CMIP6 CMIP. Retrieved: August 2022. Earth System Grid Federation. doi:https://doi.org/10.22033/ESGF/CMIP6.10205

Tang, Yongming et al. (2019). MOHC UKESM1.0-LL model output prepared for CMIP6 CMIP. Retrieved: August 2022. Earth System Grid Federation. doi:https://doi.org/10.22033/ESGF/CMIP6.1569

Volodin, Evgeny et al. (2019a). INM INM-CM4-8 model output prepared for CMIP6 CMIP. Retrieved: August 2022. Earth System Grid Federation. doi:https://doi.org/10.22033/ESGF/CMIP6.1422

Volodin, Evgeny et al. (2019b). INM INM-CM5-0 model output prepared for CMIP6 CMIP. Retrieved: August 2022 Earth System Grid Federation. doi:https://doi.org/10.22033/ESGF/CMIP6.1423

Wieners, Karl-Hermann et al. (2019). MPI-M MPIESM1.2-LR model output prepared for CMIP6 CMIP. Retrieved: August 2022. Earth System Grid Federation. doi:https://doi.org/10.22033/ESGF/CMIP6.742

Yang, T., Liu, X., Han, Z., 2022. Predicting the Effects of Climate Change on the Suitable Habitat of Japanese Spanish Mackerel (Scomberomorus niphonius) Based on the Species Distribution Model. Frontiers in Marine Science 9.

Zając, A., Zając, M., Tertil, R. & Harman, I. Atlas rozmieszczenia roślin naczyniowych w Polsce–Distribution Atlas of Vascular Plants in Poland. (Nakladem Pracowni Chorologii Komputerowej Instytutu Botaniki Uniwersytetu - Laboratory of Computer Corology - Institute of Botany - Jagiellonian University, 2001).

Ziehn, Tilo et al. (2019). CSIRO ACCESS-ESM1.5 model output prepared for CMIP6 CMIP. Retrieved: August 2022. Earth System Grid Federation. doi:https://doi.org/10.22033/ESGF/CMIP6.2288

Zurell, D., Franklin, J., König, C., Bouchet, P.J., Dormann, C.F., Elith, J., Fandos, G., Feng, X., Guillera-Arroita, G., Guisan, A., Lahoz-Monfort, J.J., Leitão, P.J., Park, D.S., Peterson, A.T., Rapacciuolo, G., Schmatz, D.R., Schröder, B., Serra-Diaz, J.M., Thuiller, W., Yates, K.L., Zimmermann, N.E., Merow, C., 2020. A standard protocol for reporting species distribution models. Ecography 43, 1261–1277. https://doi.org/10.1111/ecog.04960
